# Adherence to a Mediterranean diet is associated with a lower risk of diabetic kidney disease among individuals with hyperglycemia: a prospective cohort study

**DOI:** 10.1186/s12916-024-03455-3

**Published:** 2024-06-03

**Authors:** Changbo Qu, Jinyu Zhao, Jicai Lai, Xinxiang Wu, Peng Huang, Ting Zhu, Yan Li, Taoli Liu, Jinqiu Yuan, Ning Wang, Maikel P Peppelenbosch, Hongda Chen, Bin Xia, Jian Qin

**Affiliations:** 1 Department of Traditional Chinese Medicine, The Seventh Affiliated Hospital of Sun Yat-sen University (SAHSYSU), Shenzhen, 518107 China; 2 The First Clinical Medical School, Lanzhou University, Lanzhou, 73000 China; 3 The Fourth Clinical Medical College of Guangzhou University of Chinese Medicine, Shenzhen, 518107 China; 4 Scientific Research Center, The Seventh Affiliated Hospital, Sun Yat-sen University, Shenzhen, 518107 China; 5 Department of Nephrology, Center of Kidney and Urology, The Seventh Affiliated Hospital, Sun Yat-sen University, Shenzhen, 518107 China; 6 School of Medicine, Sun Yat-sen University, Shenzhen, 518107 China; 7 Clinical Research Center, The Seventh Affiliated Hospital, Sun Yat-Sen University, Shenzhen, 518107 China; 8 Chinese Health Risk Management Collaboration, Shenzhen, 518107 China; 9 Department of Gastroenterology and Hepatology, Erasmus MC-University Medical Center, Rotterdam, the Netherlands

**Keywords:** Mediterranean diet, Microvascular complications, UK Biobank

## Abstract

**Background:**

Type 2 diabetes is associated with a variety of complications, including micro- and macrovascular complications, neurological manifestations and poor wound healing. Adhering to a Mediterranean Diet (MED) is generally considered an effective intervention in individuals at risk for type 2 diabetes mellitus (T2DM). However, little is known about its effect with respect to the different specific manifestations of T2DM. This prompted us to explore the effect of MED on the three most significant microvascular complications of T2DM: diabetic retinopathy (DR), diabetic kidney disease (DKD), and vascular diabetic neuropathies (DN).

**Methods:**

We examined the association between the MED and the incidence of these microvascular complications in a prospective cohort of 33,441 participants with hyperglycemia free of microvascular complications at baseline, identified in the UK Biobank. For each individual, we calculated the Alternate Mediterranean Diet (AMED) score, which yields a semi-continuous measure of the extent to which an individual’s diet can be considered as MED. We used Cox proportional hazard models to analyze hazard ratios (HRs) and 95% confidence intervals (CIs), adjusting for demographics, lifestyle factors, medical histories and cardiovascular risk factors.

**Results:**

Over a median of 12.3 years of follow-up, 3,392 cases of microvascular complications occurred, including 1,084 cases of diabetic retinopathy (DR), 2,184 cases of diabetic kidney disease (DKD), and 632 cases of diabetic neuropathies (DN), with some patients having 2 or 3 microvascular complications simultaneously. After adjusting for confounders, we observed that higher AMED scores offer protection against DKD among participants with hyperglycemia (comparing the highest AMED scores to the lowest yielded an HR of 0.79 [95% CIs: 0.67, 0.94]). Additionally, the protective effect of AMED against DKD was more evident in the hyperglycemic participants with T2DM (HR, 0.64; 95% CI: 0.50, 0.83). No such effect, however, was seen for DR or DN.

**Conclusions:**

In this prospective cohort study, we have demonstrated that higher adherence to a MED is associated with a reduced risk of DKD among individuals with hyperglycemia. Our study emphasizes the necessity for continued research focusing on the benefits of the MED. Such efforts including the ongoing clinical trial will offer further insights into the role of MED in the clinical management of DKD.

**Supplementary Information:**

The online version contains supplementary material available at 10.1186/s12916-024-03455-3.

## Background

Type 2 diabetes mellitus (T2DM) is associated with a variety of complications including micro- and macrovascular complications, neurological manifestations and poor wound healing. Diabetic retinopathy (DR), diabetic kidney disease (DKD), and vascular diabetic neuropathies (DN) are the three most significant microvascular complications and contribute significantly to morbidity and mortality worldwide [1]. Among these microvascular complications, DKD is highly prevalent and stands as the leading cause of kidney failure and end-stage kidney disease [2]. Notably, microvascular complications are also observed in patients with non-diabetic hyperglycemia, where glucose metabolism is abnormal but remains below the diagnostic criteria for T2DM [3].

Glucose-lowering agents such as metformin, sodium-glucose co-transporter-2 (SGLT2) inhibitors and glucagon-like peptide 1 (GLP-1) receptor agonists, are generally recommended to ameliorate hyperglycemia [4]. However, these drugs may initially exacerbate DR and have been frequently associated with genitourinary infections, lactic acidosis and gastrointestinal side effects [5–7]. Antihypertensive drugs such as angiotensin-converting enzyme (ACE) inhibitors have been used for the treatment of DKD and DR; however, it is not well tolerated in some aged patients and may increase the risk of hyperkalemia when used in combination with angiotensin-receptor blockers (ARBs) [5]. In addition to drug treatment, diet therapy has been established as one of the most promising strategies for the management of hyperglycemia. The Mediterranean diet (MED) is a healthy dietary pattern that is characterized by increased consumption of legumes, vegetables, fruits, olive oil, whole-grain cereals and nuts. It also involves a moderate intake of fish and red wine, while limiting the consumption of red meat products and saturated fatty acids [8, 9]. In an umbrella review of meta-analyses, it has been revealed that higher adherence to the MED was associated with a 19% to 27% reduction in the risk of developing diabetes [10]. Furthermore, the MED was found to remarkably decrease the risk of T2DM in 8,291 Italian patients with a recent myocardial infarction [11] and 13,380 healthy Spanish university graduates [12].

Several systematic reviews have been conducted to investigate the impact of dietary interventions on microvascular complications. For instance, it has been shown that low-protein diets could significantly decrease urea levels in patients with DKD [13]. Additionally, adopting a vegetarian dietary pattern has been suggested to have a beneficial effect on DKD [14]. Furthermore, reducing salt intake among diabetic individuals has been found to slow the progression of DKD [15]. Previous systematic reviews have also revealed that higher adherence to the MED is associated with reduced risks of incident DR [16, 17]. However, it appears that adhering to the MED may have distinct effects on individual microvascular complications [18, 19]. Here, we sought to investigate the association between MED and the incidence of microvascular complications by performing a pooled analysis among participants with hyperglycemia. We have also investigated the effects of individual components of the MED on microvascular complications.

## Methods

### Study population

**Fig. 1:**
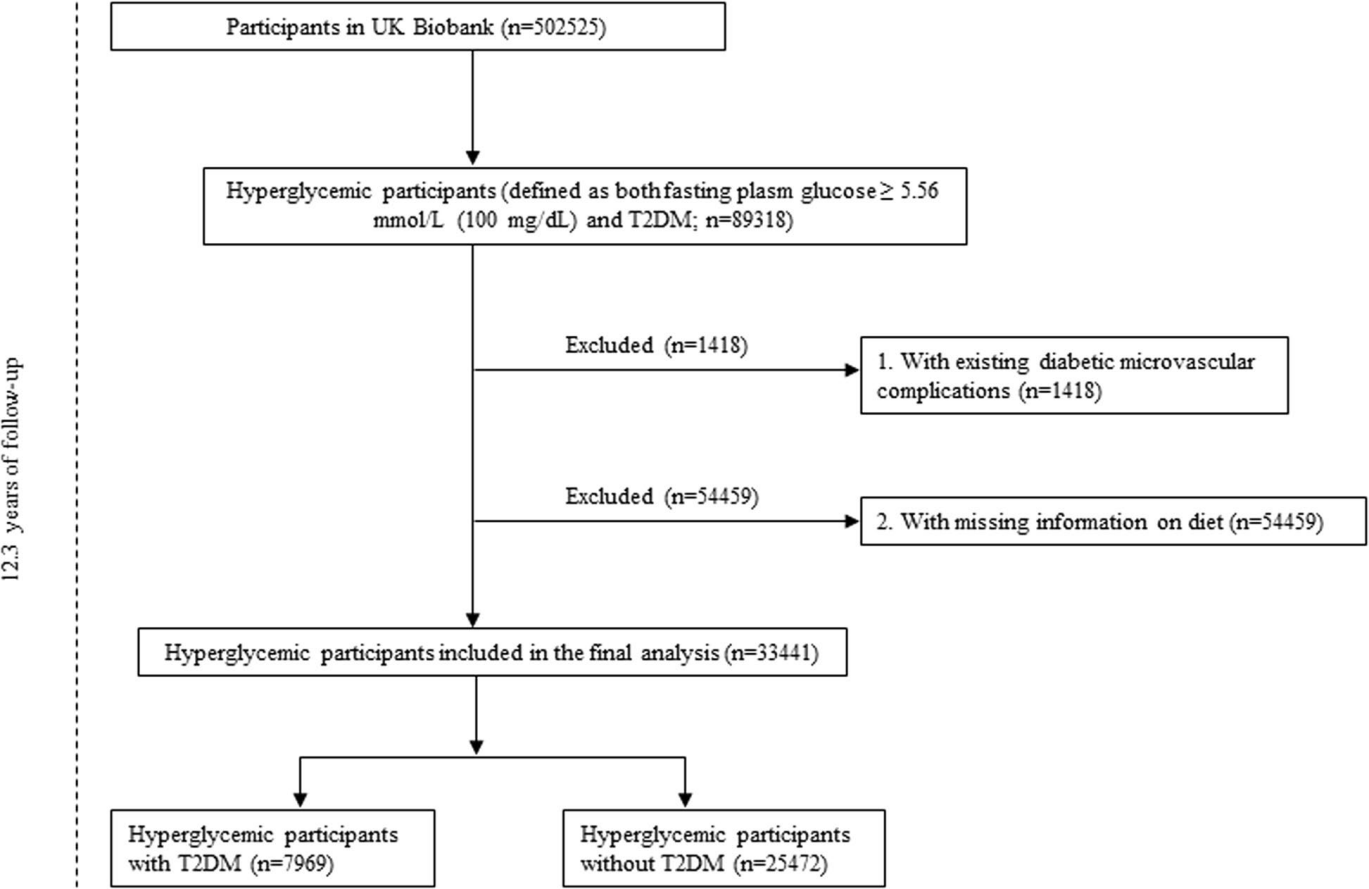
Flowchart of the selection of the study population from the UK Biobank

The UK Biobank cohort study recruited approximately 500,000 middle and aged adults across the UK from 2006 to 2010. We included hyperglycemic participants (defined as both fasting plasm glucose ≥ 5.56 mmol/L (100 mg/dL) and T2DM (self-reported or doctor-diagnosed, who were taking anti-hyperglycemic medications or insulin, or had glycosylated hemoglobin (HbA1c) levels > 48 mmol/mol)) and excluded those with a diagnosis of microvascular complications, incomplete dietary pattern information, or those who withdrew from the study (n=33441) based on previous criteria [20, 21]. The hyperglycemic participants include individuals with T2DM (n=7,969) and those without T2DM (n=25,472). The flowchart illustrating the inclusion of patients in our present study can be found in Fig. 1. The UK Biobank holds ethical approval from the North West Multi-Centre Research Ethics Committee and all participants provided informed consent. Our research protocol for this study has obtained approvals from the review committees associated with the UK Biobank.

### Dietary assessment

The Alternate Mediterranean Diet (AMED) score was slightly modified from a traditional MED score [22–24]. Briefly, the AMED classifies various food items into nine distinct groups, which include vegetables, fruits, legumes, nuts, whole grains, fish, monounsaturated-to-saturated fat ratio (MUFA to SFA ratio), red and processed meats, and alcohol consumption. The total AMED score ranged from 0 to 9, with points assigned based on dietary habits. Points were assigned for higher consumption of fruits, vegetables, nuts, legumes, whole grains, fish, MUFA to SFA ratio, moderate consumption of alcohol, and lower consumption of red/processed meat. For each of these components, a score of 0 or 1 was assigned according to whether the participant was below or above the sex-specific median level of consumption in the study population and whether the component received points for lower or higher consumption (Additional file 1: Table S1) [25].

### Assessment of main outcomes

Using the inclusion criteria provided in a recent study [21], we identified microvascular complications, including diabetic retinopathy (ICD-10: E113, E143, H280, H360), diabetic neuropathy (ICD-10: E114, E144, G590, G629, G632, G990), and diabetic kidney disease (ICD-10: E112, E142, N180, N181, N182, N183, N184, N185, N188, N189). To calculate the follow-up time, we measured the duration from the time of returning the baseline questionnaire to the occurrence of microvascular complications, the date of death, the date when a participant was lost to follow-up, or the end of the follow-up period, depending on which event occurred first.

### Assessment of covariates

All participants completed touchscreen questionnaires, providing detailed information on socio-demographic factors such as age, gender, ethnicity, and the Index of Multiple Deprivation (IMD) [26]. They also reported their lifestyle habits, including dietary intake, physical activity levels, and alcohol consumption. Medical histories were documented as well. In addition to self-reported data, anthropometric measurements, such as height, body weight, and waist circumference (WC), were taken at the assessment center. Blood samples were collected from participants who had provided their consent at the time of recruitment. These samples were carefully stored at the UK Biobank under controlled conditions until they were later analyzed for various circulating biomarkers. In our analysis, several circulating biomarkers were considered to exhibit a positive correlation with an increased risk of DKD and thus treated as potential confounding factors. These biomarkers included glycated hemoglobin (HbA1c), insulin-like growth factor 1 (IGF), total cholesterol (TC), creatinine (CRT), high-density lipoprotein cholesterol (HDL-C), low-density lipoprotein cholesterol (LDL-C), and triglycerides (TG).

### Statistical analysis

The hazard ratios (HRs) and 95% confidence intervals (CIs) were estimated using Cox regression models. Two distinct models were constructed for this analysis. In model 1, we adjusted for age (continuous, years) and gender (men or women). In model 2, we additionally adjusted for ethnicity (white or other), index of multiple deprivations (a measure of socioeconomic status) [26], waist circumference (WC (continuous, centimeters)), alcohol consumption (categorized as never or special occasions only, one to three times a month, one to four times a week, daily or almost daily), physical activity (h/week), hypertension (yes or no), family history of diabetes (yes or no), family history of hypertension (yes or no), family history of heart disease (yes or no) and family history of stroke (yes or no), HbA1c (continuous, mmol/L), HDL-C (continuous, mmol/L), LDL-C (continuous, mmol/L), IGF (continuous, mmol/L), TG (continuous, mmol/L), CHOL (continuous, mmol/L), use of antihypertensive medication, use of cholesterol-lowering medication or use of diabetes medication (yes or no). Restricted cubic spline analysis was used to investigate the dose-response associations between the healthy lifestyle score and risks of microvascular complications. In the sensitivity analyses, we performed a lagged analysis of the exposure for 2 or 4 years, which could strengthen the temporality and allow a time window for the development of microvascular complications. The creatinine levels (continuous, µmol/L) and frequency of meat intake (times/week) were further adjusted for participants diagnosed with DKD. To investigate potential effect modifiers, we conducted stratified analyses according to age, gender, IMD, WC, alcohol intake, HbA1c, HDL-C, LDL-C, IGF, TG, TC, hypertension, physical activity, and use of antidiabetic medication, cholesterol-lowering medication and antihypertensive medication.

## Results

The characteristics of study participants at baseline were listed according to their AMED scores. Among the 25,472 hyperglycemic participants without T2DM (48.9% men; mean age, 58.36 years), there were 2,495 (9.8%), 4,425 (17.4%), 5,176 (20.3%), 4,878 (19.2%), 4,194 (16.5%), 4,304 (16.9%) having 0-1, 2, 3, 4, 5, and 6-9 AMED score. Participants with higher AMED scores appear to be older, female and have lower levels of WC, IMD, and HbA1c. Additionally, they were less likely to use medications for hyperlipemia and hypertension (Table 1). Among the 7,969 hyperglycemic participants with T2DM (63.9% men; mean age, 60.28 years), there were 917 (11.5%), 1,573 (19.7%), 1,825 (22.9%), 1,464 (18.4%), 1,166 (14.6%), 1,024 (12.8%) having 0-1, 2, 3, 4, 5, and 6-9 AMED score. Participants with higher AMED scores also have lower levels of WC, IMD, and HbA1c (Table 2).

**Table 1:** Baseline characteristics of the hyperglycemic participants without T2DM according to the numbers of Alternate Mediterranean Diet (AMED)

	Numbers of AMED							P value
	Total	0-1	2	3	4	5	6-9	
Number of patients	25472	2495	4425	5176	4878	4194	4304	
Age, years	58.36 ± 7.58	57.92 ± 7.80	57.88 ± 7.77	58.14 ± 7.65	58.46 ± 7.58	58.92 ± 7.26	58.71 ± 7.42	<0.001
Men (%)	12448 (48.9)	1321 (52.9)	2341 (52.9)	2561 (49.5)	2345 (48.1)	1995 (47.6)	1885 (43.8)	<0.001
Ethnicity, White (%)	24325 (95.5)	2385 (95.6)	4203 (95.0)	4889 (94.5)	4662 (95.6)	4012 (95.7)	4174 (97.0)	<0.001
Index of Multiple Deprivation	12.31 [6.99, 20.94]	13.36 [7.74, 23.02]	13.31 [7.31, 22.64]	12.61 [7.05, 21.59]	11.91 [6.84, 20.40]	11.88 [6.77, 20.21]	11.30 [6.70, 18.75]	<0.001
Waist circumference, cm	92.99 ± 14.05	95.52 ± 13.81	94.67 ± 14.03	93.45 ± 13.78	92.64 ± 13.99	92.07 ± 13.82	90.51 ± 14.32	<0.001
Never drinker (%)	4462 (17.5)	460 (18.4)	850 (19.2)	955 (18.5)	837 (17.2)	725 (17.3)	635 (14.8)	<0.001
HbA1c, mmol/L	37.92 ± 6.29	38.35 ± 6.36	38.18 ± 6.35	37.98 ± 6.45	37.80 ± 6.36	37.90 ± 6.45	37.48 ± 5.70	<0.001
HDL-C, mmol/L	1.43 ± 0.39	1.39 ± 0.37	1.40 ± 0.38	1.43 ± 0.39	1.44 ± 0.40	1.45 ± 0.38	1.46 ± 0.39	<0.001
LDL-C, mmol/L	3.56 ± 0.88	3.58 ± 0.88	3.56 ± 0.88	3.58 ± 0.88	3.56 ± 0.87	3.54 ± 0.88	3.53 ± 0.85	0.023
IGF, mmol/L	21.23 ± 5.79	20.70 ± 5.66	20.93 ± 5.59	21.23 ± 6.03	21.28 ± 6.12	21.38 ± 5.46	21.65 ± 5.63	<0.001
TG, mmol/L	1.93 ± 1.12	2.00 ± 1.08	2.02 ± 1.19	1.95 ± 1.11	1.93 ± 1.15	1.87 ± 1.05	1.85 ± 1.10	<0.001
CHOL, mmol/L	5.70 ± 1.16	5.69 ± 1.17	5.68 ± 1.17	5.72 ± 1.17	5.71 ± 1.17	5.68 ± 1.17	5.68 ± 1.14	0.231
Use of cholesterol lowering medication (%)	5737 (22.5)	595 (23.8)	1074 (24.3)	1175 (22.7)	1052 (21.6)	947 (22.6)	894 (20.8)	0.001
Hypertension (%)	20847 (81.8)	2036 (81.6)	3642 (82.3)	4296 (83.0)	3987 (81.7)	3420 (81.5)	3466 (80.5)	0.058
Use of antihypertensive medication (%)	7105 (27.9)	747 (29.9)	1266 (28.6)	1485 (28.7)	1344 (27.6)	1150 (27.4)	1113 (25.9)	0.004
Family history of diabetes (%)	6568 (25.8)	687 (27.5)	1226 (27.7)	1301 (25.1)	1263 (25.9)	1044 (24.9)	1047 (24.3)	0.001
Family history of hypertension (%)	12814 (50.3)	1242 (49.8)	2173 (49.1)	2607 (50.4)	2446 (50.1)	2140 (51.0)	2206 (51.3)	0.381
Family history of heart disease (%)	11594 (45.5)	1110 (44.5)	1932 (43.7)	2320 (44.8)	2234 (45.8)	1943 (46.3)	2055 (47.7)	0.003
Family history of stroke (%)	6958 (27.3)	667 (26.7)	1197 (27.1)	1373 (26.5)	1365 (28.0)	1208 (28.8)	1148 (26.7)	0.114
Physical activity (MET), h/week	27.40 [12.55, 53.77]	25.20 [10.47, 49.52]	25.77 [11.62, 52.87]	27.50 [12.00, 56.75]	26.63 [12.55, 51.08]	28.60 [13.55, 56.35]	29.90 [14.22, 55.12]	<0.001

Data at baseline are presented as mean ± SD, median [25th, 75th percentiles], or n (%)

**Table 2:** Baseline characteristics of the hyperglycemic participants with T2DM according to the numbers of Alternate Mediterranean Diet (AMED)

	Numbers of AMED							*P* value
	Total	0-1	2	3	4	5	6-9	
Number of patients	7969	917	1573	1825	1464	1166	1024	
Age, years	60.28 ± 6.73	60.21 ± 6.82	60.03 ± 6.83	60.14 ± 6.88	60.29 ± 6.69	60.37 ± 6.58	60.83 ± 6.41	0.07
Men (%)	5090 (63.9)	591 (64.4)	1001 (63.6)	1182 (64.8)	946 (64.6)	753 (64.6)	617 (60.3)	0.205
Ethnicity, White (%)	7198 (90.3)	839 (91.5)	1399 (88.9)	1641 (89.9)	1320 (90.2)	1073 (92.0)	926 (90.4)	0.103
Index of Multiple Deprivation	14.61 [8.04, 25.32]	14.83 [8.76, 25.06]	15.57 [8.24, 28.05]	15.21 [8.31, 26.96]	14.91 [8.11, 24.99]	13.53 [7.80, 23.25]	13.05 [7.35, 21.78]	<0.001
Waist circumference, cm	103.35 (14.16)	104.45 (13.78)	103.72 (14.07)	103.65 (14.21)	103.02 (14.16)	103.26 (14.14)	101.85 (14.45)	0.001
Never drinker (%)	2363 (29.7)	274 (29.9)	517 (32.9)	540 (29.6)	441 (30.1)	329 (28.2)	262 (25.6)	0.004
HbA1c, mmol/L	51.99 ± 13.44	52.45 ± 13.82	52.15 ± 13.31	52.69 ± 14.10	51.29 ± 12.97	52.02 ± 13.49	51.02 ± 12.57	0.008
HDL-C, mmol/L	1.22 ± 0.30	1.22 ± 0.30	1.23 ± 0.31	1.22 ± 0.31	1.22 ± 0.30	1.22 ± 0.30	1.24 ± 0.30	0.379
LDL-C, mmol/L	2.82 ± 0.82	2.84 ± 0.82	2.84 ± 0.82	2.85 ± 0.83	2.76 ± 0.80	2.79 ± 0.85	2.80 ± 0.80	0.016
IGF, mmol/L	20.08 ± 6.25	19.55 ± 6.20	19.98 ± 6.79	19.78 ± 5.72	20.41 ± 6.23	20.20 ± 6.37	20.63 ± 6.17	<0.001
TG, mmol/L	2.18 ± 1.24	2.20 ± 1.21	2.18 ± 1.23	2.20 ± 1.21	2.17 ± 1.33	2.17 ± 1.15	2.18 ± 1.26	0.968
CHOL, mmol/L	4.66 ± 1.11	4.68 ± 1.10	4.68 ± 1.10	4.69 ± 1.12	4.60 ± 1.12	4.63 ± 1.14	4.66 ± 1.09	0.134
Use of cholesterol lowering medication (%)	5803 (72.8)	654 (71.3)	1134 (72.1)	1315 (72.1)	1093 (74.7)	862 (73.9)	745 (72.8)	0.388
Hypertension (%)	7303 (91.6)	838 (91.4)	1434 (91.2)	1687 (92.4)	1347 (92.0)	1068 (91.6)	929 (90.7)	0.631
Use of antihypertensive medication (%)	4864 (61.0)	543 (59.2)	999 (63.5)	1106 (60.6)	899 (61.4)	712 (61.1)	605 (59.1)	0.206
Use of diabetes medication (%)	5000 (62.7)	579 (63.1)	1017 (64.7)	1166 (63.9)	901 (61.5)	738 (63.3)	599 (58.5)	0.029
Family history of diabetes (%)	3589 (45.0)	396 (43.2)	735 (46.7)	794 (43.5)	652 (44.5)	535 (45.9)	477 (46.6)	0.280
Family history of hypertension (%)	4128 (51.8)	452 (49.3)	813 (51.7)	912 (50.0)	776 (53.0)	634 (54.4)	541 (52.8)	0.103
Family history of heart disease (%)	3941 (49.5)	460 (50.2)	752 (47.8)	841 (46.1)	750 (51.2)	594 (50.9)	544 (53.1)	0.002
Family history of stroke (%)	2423 (30.4)	281 (30.6)	459 (29.2)	526 (28.8)	444 (30.3)	377 (32.3)	336 (32.8)	0.148
Physical activity (MET), h/week	22.40 [8.78, 46.77]	23.51 [8.63, 51.04]	20.80 [8.12, 44.36]	20.92 [9.14, 45.50]	21.87 [8.97, 47.12]	23.10 [9.03, 46.22]	24.10 [9.78, 48.88]	0.085

Data at baseline are presented as mean ± SD, median [25th, 75th percentiles], or n (%)

**Fig. 2:**
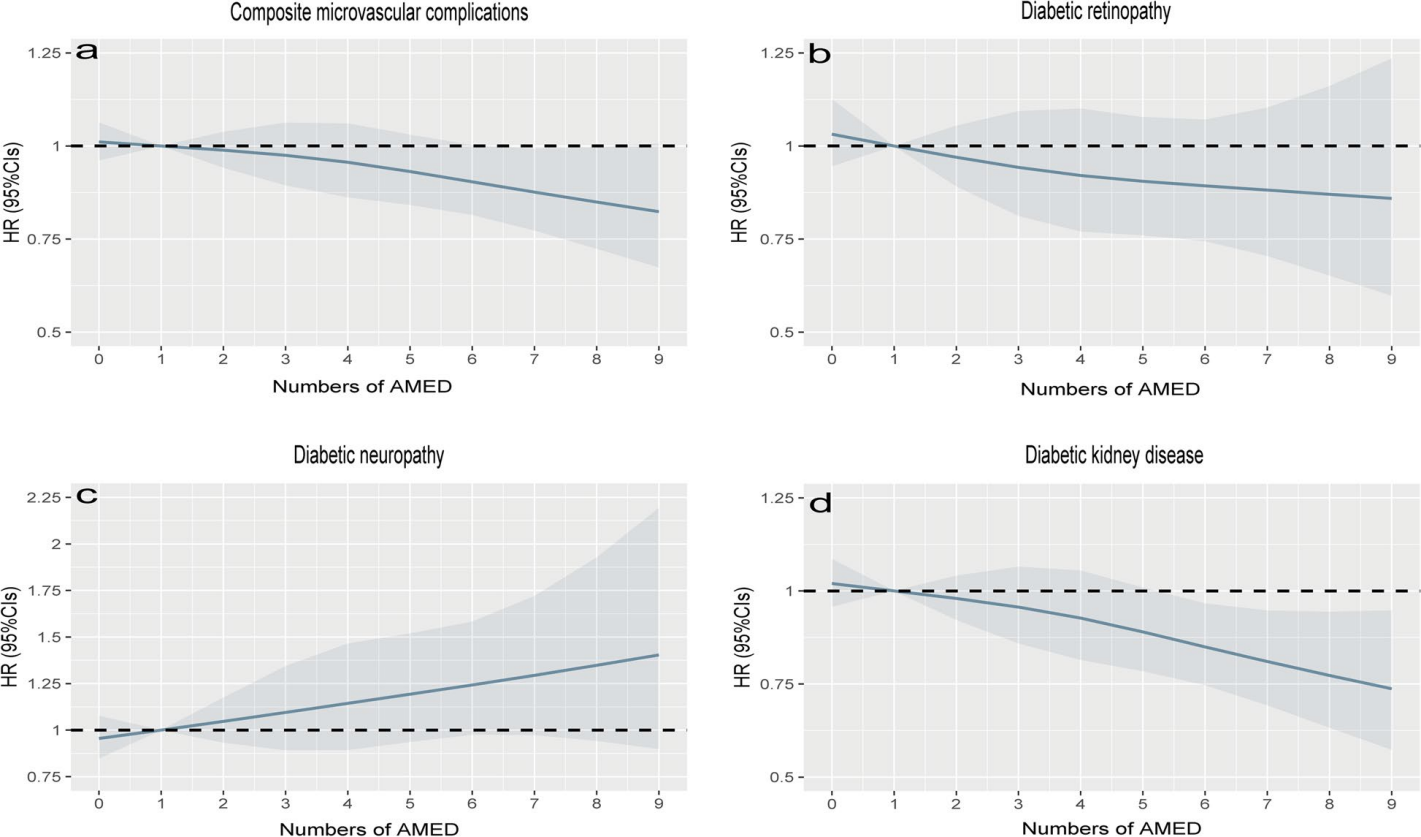
Dose-response relationship of AMED with the risks of microvascular complications among total participants with hyperglycemia. The X-axis showed the numbers of AMED, and the y-axis showed the HRs of the composite microvascular complications (**a**), diabetic retinopathy (**b**), diabetic neuropathy (**c**), and diabetic kidney disease (**d**). Multivariable-adjusted models were adjusted for age (continuous, years) and gender (men or women), ethnicity (white or other), index of multiple deprivation (a measure of socioeconomic status), waist circumference (continuous, centimeters), alcohol consumption (categorized as never or special occasions only, one to three times a month, one to four times a week, daily or almost daily), physical activity (h/week), hypertension (yes or no), family history of diabetes (yes or no), family history of hypertension (yes or no), family history of heart disease(yes or no) and family history of stroke (yes or no), HbA1c (continuous, mmol/L), HDL-C (continuous, mmol/L), LDL-C (continuous, mmol/L), IGF (continuous, mmol/L), TG (continuous, mmol/L), CHOL (continuous, mmol/L), use of antihypertensive medication, cholesterol lowering medication and diabetes medication

**Fig. 3:**
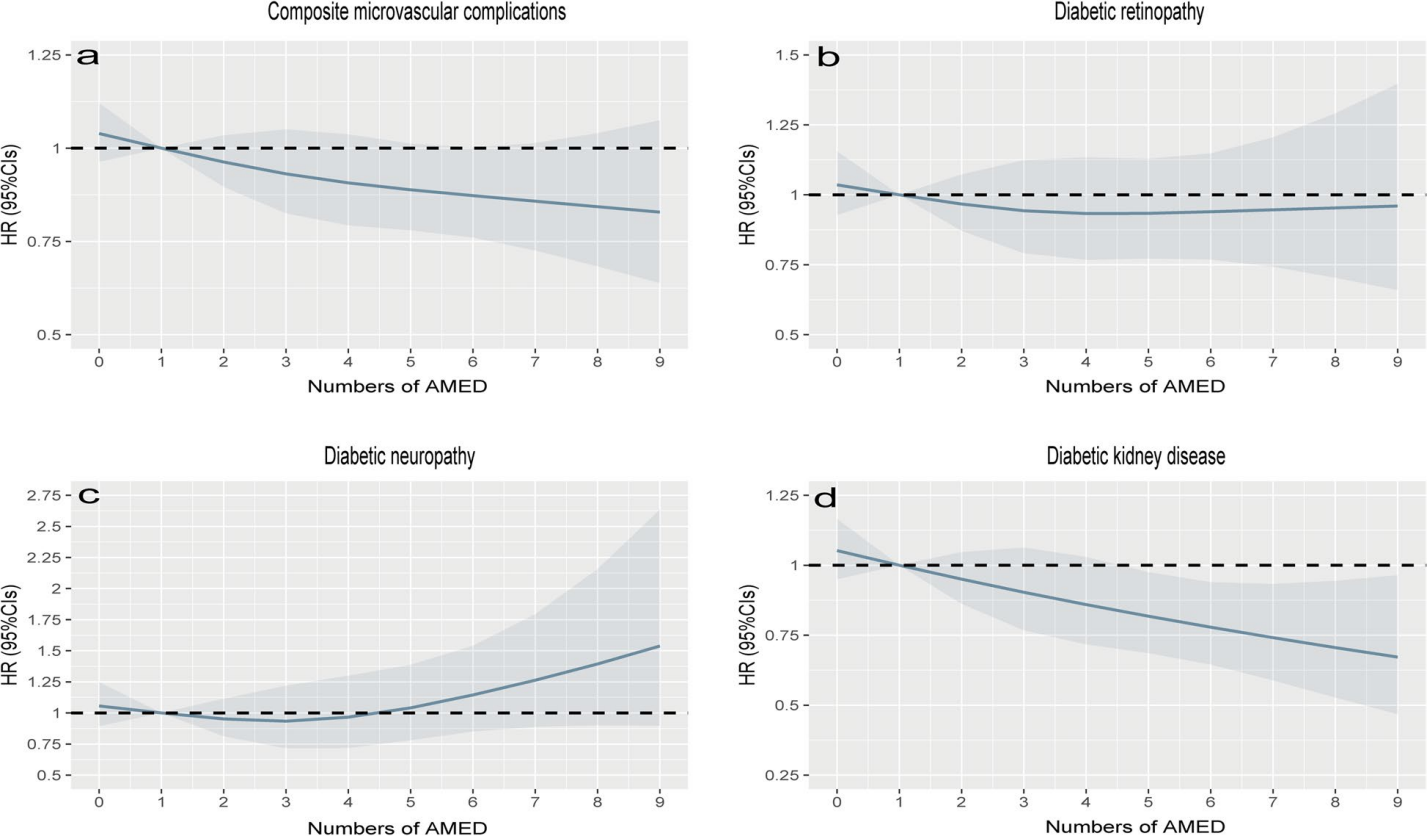
Dose-response relationship of AMED with the risks of microvascular complications among hyperglycemic participants with T2DM. The X-axis showed the numbers of AMED, and the y-axis showed the HRs of the composite microvascular complications (**a**), diabetic retinopathy (**b**), diabetic neuropathy (**c**), and diabetic kidney disease (**d**). Multivariable-adjusted models were adjusted for age (continuous, years) and gender (men or women), ethnicity (white or other), index of multiple deprivation (a measure of socioeconomic status), waist circumference (continuous, centimeters), alcohol consumption (categorized as never or special occasions only, one to three times a month, one to four times a week, daily or almost daily), physical activity (h/week), hypertension (yes or no), family history of diabetes (yes or no), family history of hypertension (yes or no), family history of heart disease(yes or no) and family history of stroke (yes or no), HbA1c (continuous, mmol/L), HDL-C (continuous, mmol/L), LDL-C (continuous, mmol/L), IGF (continuous, mmol/L), TG (continuous, mmol/L), CHOL (continuous, mmol/L), use of antihypertensive medication, cholesterol lowering medication and diabetes medication

Among the total of 33,441 participants with hyperglycemia, there were 3,412 (10.2%), 5,998 (17.9%), 7,001 (20.9%), 6,342 (18.9%), 5,360 (16%), 5,328 (15.9%) having 0-1, 2, 3, 4, 5, and 6-9 AMED score (Table 3). During 404,918 person-years of follow-up (median 12.3 years), a total of 3,392 cases of composite microvascular complications were observed. These complications consisted of 1,084 cases of DR, 632 cases of DN, and 2,184 cases of DKD, with some patients being diagnosed with multiple microvascular complications simultaneously. After correcting for the confounding factors, the multivariable analyses showed that there were inverse associations between overall AMED scores and the risk of DKD; however, no such association was seen for DR and DN (Table 3 and Fig. 2). Moreover, comparing the highest AMED scores to the lowest yielded an HR of 0.79 [95% CIs: 0.67, 0.94] for DKD in participants with hyperglycemia (Table 3), indicating that higher AMED adherence protects DKD. Next, we conducted separate analyses for hyperglycemic participants with and without T2DM. The results revealed that adherence to AMED offers greater protection against DKD in the hyperglycemic participants with T2DM (HR, 0.64; 95% CI: 0.5, 0.83) (Table 4 and Fig. 3), whereas a reduced protective effect was observed in those without T2DM (Additional file 1: Table S2 and Additional file 1: Fig. S1).

**Table 3:** HRs (95% CIs) of microvascular complications according to the numbers of AMED among total participants with hyperglycemia

	Numbers of AMED							
	0-1	2	3	4	5	6-9	P _trend_	HR _continuous_
Number of patients	3412	5998	7001	6342	5360	5328		
Composite microvascular complications								
Cases/Person-years	388/40858	662/72187	753/84520	638/76954	512/65062	439/65337		
Unadjusted	1.00	0.97 (0.86, 1.10)	0.93 (0.82, 1.05)	0.85 (0.75, 0.97)	0.79 (0.70, 0.91)	0.68 (0.59, 0.78)	<0.001	0.93 (0.91, 0.95)
Model 1	1.00	0.97 (0.85, 1.10)	0.94 (0.83, 1.06)	0.86 (0.76, 0.98)	0.8 (0.70, 0.92)	0.69 (0.61, 0.80)	<0.001	0.93 (0.91, 0.95)
Model 2	1.00	0.98 (0.87, 1.12)	0.97 (0.86, 1.10)	0.96 (0.85, 1.09)	0.92 (0.81, 1.05)	0.87 (0.75, 0.99)	0.019	0.98 (0.96, 0.99)
Diabetic retinopathy								
Cases/Person-years	129/42173	230/74151	238/86984	182/79114	177/66708	128/66771		
Unadjusted	1.00	1.02 (0.82, 1.26)	0.89 (0.72, 1.10)	0.74 (0.59, 0.93)	0.84 (0.67, 1.05)	0.61 (0.48, 0.78)	<0.001	0.91 (0.88, 0.95)
Model 1	1.00	1.02 (0.82, 1.26)	0.89 (0.72, 1.11)	0.75 (0.60, 0.94)	0.85 (0.68, 1.07)	0.63 (0.49, 0.80)	<0.001	0.92 (0.88, 0.95)
Model 2	1.00	1.07 (0.86, 1.33)	0.93 (0.75, 1.16)	0.87 (0.69, 1.09)	1.04 (0.83, 1.31)	0.86 (0.67, 1.10)	0.179	0.98 (0.94, 1.01)
Diabetic neuropathy								
Cases/Person-years	64/42427	116/74755	136/87451	130/79291	89/67031	97/66869		
Unadjusted	1.00	1.03 (0.76, 1.40)	1.03 (0.76, 1.38)	1.07 (0.79, 1.44)	0.86 (0.62, 1.18)	0.94 (0.68, 1.29)	0.306	0.98 (0.93, 1.02)
Model 1	1.00	1.03 (0.76, 1.40)	1.04 (0.77, 1.40)	1.09 (0.81, 1.48)	0.88 (0.64, 1.21)	0.98 (0.72, 1.35)	0.527	0.99 (0.94, 1.03)
Model 2	1.00	1.05 (0.77, 1.42)	1.09 (0.81, 1.46)	1.25 (0.92, 1.68)	1.05 (0.76, 1.44)	1.32 (0.96, 1.81)	0.092	1.04 (0.99, 1.09)
Diabetic kidney disease								
Cases/Person-years	258/41569	417/73449	494/85788	413/78132	324/66086	278/66151		
Unadjusted	1.00	0.92 (0.79, 1.08)	0.92 (0.79, 1.07)	0.83 (0.71, 0.97)	0.75 (0.64, 0.89)	0.64 (0.54, 0.76)	<0.001	0.93 (0.90, 0.95)
Model 1	1.00	0.92 (0.79, 1.07)	0.92 (0.80, 1.07)	0.83 (0.71, 0.98)	0.76 (0.65, 0.90)	0.66 (0.55, 0.78)	<0.001	0.93 (0.91, 0.95)
Model 2	1.00	0.92 (0.79, 1.07)	0.96 (0.82, 1.11)	0.92 (0.79, 1.08)	0.86 (0.73, 1.01)	0.79 (0.67, 0.94)	0.005	0.97 (0.94, 0.99)

Model 1: age (continuous, years) and gender (men, women)

Model 2: Model 1+ ethnicity (white or other), index of multiple deprivation (a measure of socioeconomic status), waist circumference (continuous, in centimeters), alcohol consumption (categorized as never or special occasions only, one to three times a month, one to four times a week, daily or almost daily), physical activity (h/week), hypertension (yes or no), family history of diabetes (yes or no), family history of hypertension (yes or no), family history of heart disease(yes or no) and family history of stroke (yes or no), HbA1c (continuous, in mmol/L), HDL-C (continuous, in mmol/L), LDL-C (continuous, in mmol/L), IGF (continuous, in mmol/L), TG (continuous, in mmol/L), CHOL (continuous, in mmol/L), use of antihypertensive medication, use of cholesterol lowering medication and use of diabetes medication. Composite microvascular complications refer to the development of any types of microvascular complications, including diabetic retinopathy, diabetic kidney disease, and diabetic neuropathy. We calculated person-years for these composite microvascular complications from the date of recruitment to the date of death, diagnosis of any microvascular complications, loss to follow-up, or the end of the follow-up period, whichever occurred first. The person-years for each specific outcome were computed individually without censoring other types of microvascular complications. CI, confidence interval

**Table 4:** HRs (95% CIs) of microvascular complications according to the numbers of AMED among hyperglycemic participants with T2DM

	Numbers of AMED							
	0-1	2	3	4	5	6-9	P _trend_	HR _continuous_
Number of patients	917	1573	1825	1464	1166	1024		
Composite microvascular complications								
Cases/Person-years	250/10172	389/17723	435/20794	365/16632	283/13194	217/11933		
Unadjusted	1.00	0.90 (0.76, 1.05)	0.85 (0.72, 0.99)	0.89 (0.76, 1.04)	0.86 (0.73, 1.02)	0.71 (0.59, 0.85)	0.002	0.96 (0.93, 0.98)
Model 1	1.00	0.89 (0.76, 1.05)	0.85 (0.72,0.99)	0.88 (0.75, 1.04)	0.86 (0.72, 1.02)	0.71 (0.59, 0.85)	0.001	0.96 (0.93, 0.98)
Model 2	1.00	0.89 (0.76, 1.04)	0.85 (0.73, 0.99)	0.92 (0.78, 1.08)	0.88 (0.74, 1.04)	0.78 (0.65, 0.93)	0.039	0.97 (0.95, 1.00)
Diabetic retinopathy								
Cases/Person-years	111/10945	191/18696	203/21975	149/17716	146/13966	104/12467		
Unadjusted	1.00	1.01 (0.80, 1.28)	0.91 (0.72, 1.14)	0.83 (0.65, 1.06)	1.02 (0.80, 1.31)	0.80 (0.61, 1.05)	0.128	0.97 (0.93, 1.01)
Model 1	1.00	1.01 (0.80,1.28)	0.91 (0.72, 1.15)	0.82 (0.64, 1.05)	1.02 (0.80, 1.31)	0.80 (0.61, 1.05)	0.121	0.97 (0.93, 1.01)
Model 2	1.00	1.02 (0.81, 1.29)	0.91 (0.72, 1.14)	0.87 (0.68, 1.11)	1.06 (0.83, 1.36)	0.88 (0.67, 1.15)	0.489	0.99 (0.95, 1.03)
Diabetic neuropathy								
Cases/Person-years	49/11203	80/19312	85/22513	79/18022	58/14332	56/12694		
Unadjusted	1.00	0.95 (0.67, 1.36)	0.87 (0.61, 1.23)	1.00 (0.70, 1.43)	0.92 (0.63, 1.35)	0.99 (0.67, 1.45)	0.938	1.00 (0.95, 1.07)
Model 1	1.00	0.95 (0.67, 1.36)	0.87 (0.61, 1.23)	1.00 (0.70, 1.42)	0.92 (0.63, 1.34)	1.00 (0.68, 1.47)	0.905	1.01 (0.95, 1.07)
Model 2	1.00	0.93 (0.65, 1.33)	0.87 (0.61, 1.23)	1.05 (0.74, 1.51)	0.96 (0.66, 1.41)	1.16 (0.79, 1.71)	0.309	1.04 (0.98, 1.10)
Diabetic kidney disease								
Cases/Person-years	148/10734	201/18757	245/21748	210/17482	148/13961	109/12474		
Unadjusted	1.00	0.78 (0.63, 0.97)	0.81 (0.66, 0.99)	0.86 (0.70, 1.06)	0.76 (0.60, 0.95)	0.60 (0.47, 0.77)	0.001	0.94 (0.90, 0.97)
Model 1	1.00	0.78 (0.63, 0.96)	0.81 (0.66, 0.99)	0.86 (0.70, 1.06)	0.75 (0.60, 0.95)	0.60 (0.47, 0.77)	0.001	0.94 (0.90, 0.97)
Model 2	1.00	0.76 (0.61, 0.94)	0.81 (0.66, 0.99)	0.86 (0.70, 1.06)	0.76 (0.60, 0.95)	0.64 (0.50, 0.83)	0.010	0.95 (0.91, 0.99)

Model 1: age (continuous, years) and gender (men, women)

Model 2: Model 1+ ethnicity (white or other), index of multiple deprivation (a measure of socioeconomic status), waist circumference (continuous, in centimeters), alcohol consumption (categorized as never or special occasions only, one to three times a month, one to four times a week, daily or almost daily), physical activity (h/week), hypertension (yes or no), family history of diabetes (yes or no), family history of hypertension (yes or no), family history of heart disease (yes or no) and family history of stroke (yes or no), HbA1c (continuous, in mmol/L), HDL-C (continuous, in mmol/L), LDL-C (continuous, in mmol/L), IGF (continuous, in mmol/L), TG (continuous, in mmol/L), CHOL (continuous, in mmol/L), use of antihypertensive medication, use of cholesterol lowering medication and use of diabetes medication. Composite microvascular complications refer to the development of any types of microvascular complications, including diabetic retinopathy, diabetic kidney disease, and diabetic neuropathy. We calculated person-years for these composite microvascular complications from the date of recruitment to the date of death, diagnosis of any microvascular complications, loss to follow-up, or the end of the follow-up period, whichever occurred first. The person-years for each specific outcome were computed individually without censoring other types of microvascular complications. CI, confidence interval

**Fig. 4:**
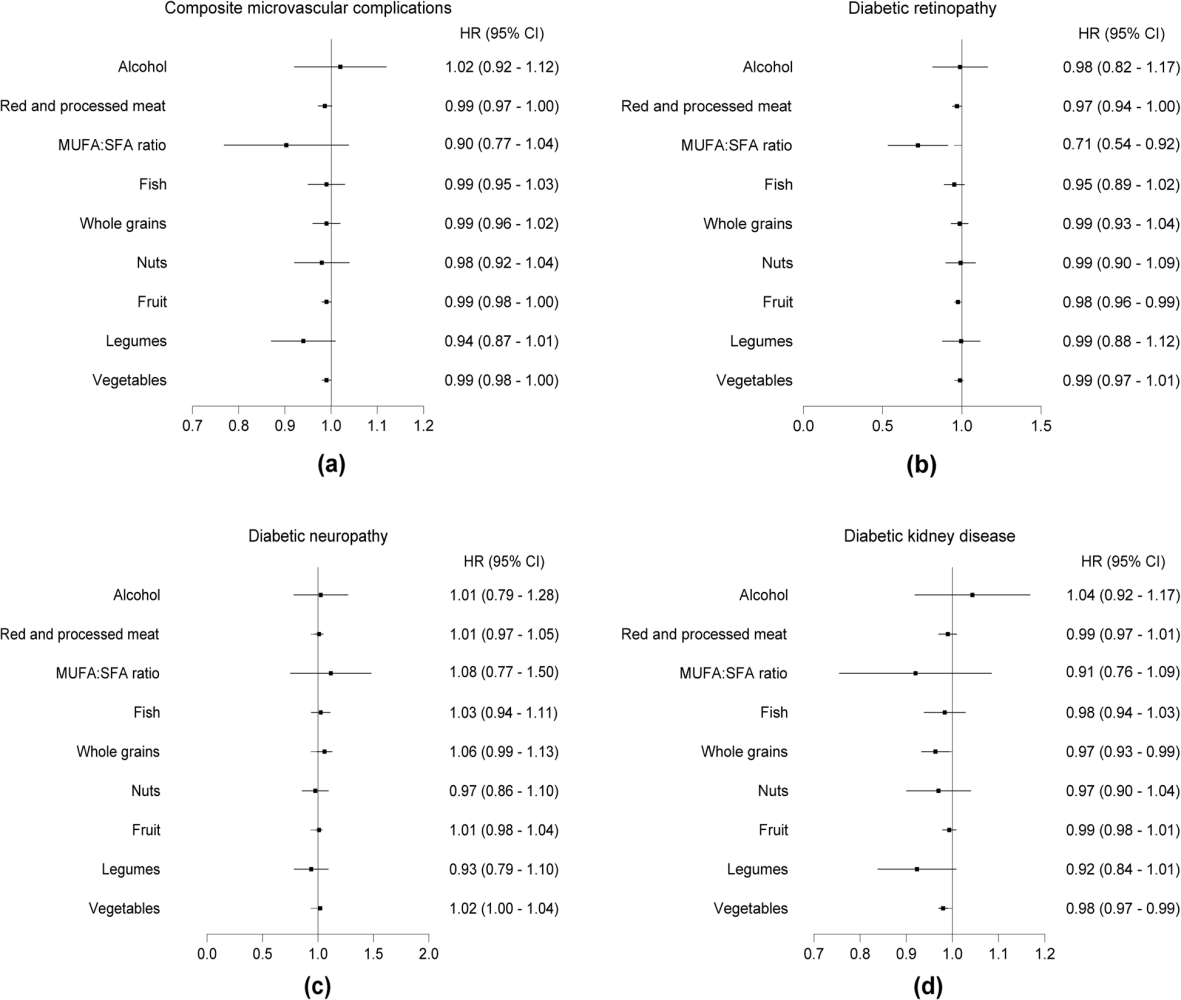
The association between adherence to individual components of AMED and the risks of microvascular complications among total participants with hyperglycemia. One point was given for intakes above the median for fruit and vegetables, legumes and nuts, whole grains and fish. In addition, one point was given for intakes below the median of red and processed meat, for use of olive or rapeseed oil for cooking or as dressing and for moderate alcohol consumption with an average of 5–15 g of alcohol per day

**Fig. 5:**
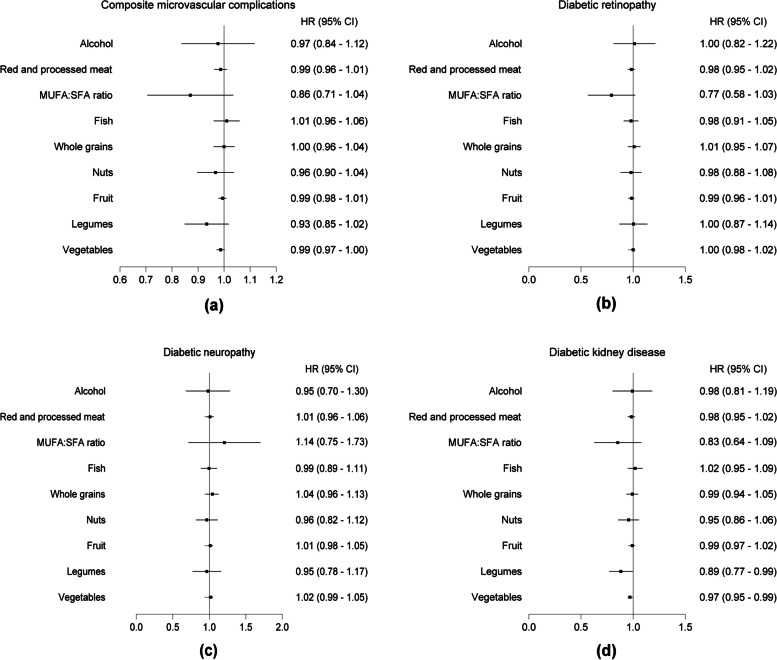
The association between adherence to individual components of AMED and the risks of microvascular complications among hyperglycemic participants with T2DM. One point was given for intakes above the median for fruit and vegetables, legumes and nuts, whole grains and fish. In addition, one point was given for intakes below the median of red and processed meat, for use of olive or rapeseed oil for cooking or as dressing and for moderate alcohol consumption with an average of 5–15 g of alcohol per day

We further investigated the associations between individual components of AMED and the risks of DKD, DR or DN. For each incremental increase in the consumption of legumes, there was a potential 8% reduction in the risk of DKD among participants with hyperglycemia (HR, 0.92; 95% CI: 0.84, 1.01) (Fig. 4). This reduction was further strengthened among hyperglycemic participants with T2DM (HR, 0.89; 95% CI: 0.77, 0.99) (Fig. 5). In the case of the MUFA: SFA ratio, there was a 29% (HR, 0.71; 95% CI: 0.54-0.92) and a potential 23% (HR, 0.77; 95% CI: 0.58-1.03) decrease in the risk of DR among these two participant groups, respectively (Figs. 4 and 5). However, we did not observe a significant reduction in the risk associated with each incremental increase in the consumption of any AMED component about DN in both groups (Figs. 4 and 5). Interestingly, whole grains and fish appear to be the main components providing benefits in decreasing the risk of DKD among hyperglycemic participants without T2DM (Additional file 1: Fig. S2).

Next, we performed subgroup analyses. For participants with hyperglycemia, the results showed that the estimates for DKD, DR or DN did not differ by gender, IMD, WC, alcohol consumption, physical activity, hypertension, use of antihypertensive medication, cholesterol-lowering medication and diabetes medication, and levels of HbA1c, HDL-C, LDL-C, IGF, TG and TC. However, the risk of DKD was lower among participants with the age of ≤ 55 years (P = 0.001) (Additional file 1: Table S3). For hyperglycemic participants with T2DM, the risk of DKD was also lower among those aged ≤ 55 years (P = 0.006) (Additional file 1: Table S4). The risk estimates for DKD, DR, or DN remained consistent among hyperglycemic participants without T2DM, regardless of these confounding factors (Additional file 1: Table S5).

Next, we performed sensitivity analyses by excluding cases that occurred within 2 or 4 years of follow-up. We observed similar trends in the associations between AMED score and the risks of microvascular complications within both a single study group and across separate groups, after lagging the exposure for 2 or 4 years (Additional file 1: Table S6-S8). To further investigate potential confounding variables that might impact the development of DKD, we conducted additional adjustments for the frequency of meat intake and creatinine levels. Our analysis revealed that adjusting for these variables did not result in significant changes in the risk of DKD (Additional file 1: Table S9).

## Discussion

Accumulating studies have evaluated the association of MED with the incidence of T2DM. For example, a recent systematic review and meta-analysis showed that adherence to the MED was significantly associated with a reduced risk of T2DM. Specifically, for each one-point increase in the MED score, there was a 3% decrease in the risk of T2DM [27]. In a three-arm randomized trial, it was also found that higher adherence to the MED was inversely associated with the risk of T2DM. Following a median follow-up of 4.0 years, the incidences of T2DM were 10.1% (95% CI: 5.1-15.1) in the MED group and 17.9% (95% CI: 11.4-24.4) in the control group [28]. Moreover, the ATTICA study, conducted among the residents of Greece's Attica province, has revealed that diabetic participants with higher MED scores had a 27% reduction in HOMA-IR, a key parameter used to assess insulin resistance [29]. Most recently, it has been reported that higher adherence to Mediterranean lifestyles could help prevent T2DM in the British adult population. This suggests a potential applicability of the MED to the non-Mediterranean populations [30].

Despite the potentially positive effect of MED in reducing the risk of T2DM, the influence of MED on microvascular complications remains inconclusive and warrants further investigation. A previous systematic review indicated that higher adherence to the MED was associated with a reduced risk of incident DR. However, the majority of the studies analyzed were cross-sectional [17]. Therefore, more longitudinal studies are needed to clarify this association. A longitudinal study conducted among 71,392 adults with diabetes in Iran showed a significant association between MED adherence and a decreased incidence of microvascular complications. However, this association was evaluated using pooled logistic regression models, which do not include time-to-event analysis [31]. A case-control study has revealed that T2DM patients consuming moderate or high MED had 62% or 86% lower risks of developing DKD compared to those with low adherence [32]. However, it is important to note that this study was exclusively conducted among women and the number of participants was relatively small, which might lead to a low statistical power. More importantly, some studies have even reported that the MED is not associated with DKD. A cross-sectional study showed that adherence to MED was not significantly associated with renal function among patients with DKD [33]. A post hoc analysis of a cohort of patients with T2DM has revealed that MED enriched with either olive oil or nuts did not exert a protective effect against DKD [18].

In this study, we demonstrated that adherence to the MED is associated with a lower risk of DKD. This aligns with previous evidence indicating that the MED can improve renal function. The Leontio Lyceum Albuminuria Study reported that adolescents with a higher adherence to MED showed decreased levels of albuminuria [34]. Additionally, a cross-sectional analysis conducted among a cohort of patients with T2DM in Taiwan demonstrated that a high intake of fish and vegetables, as opposed to the traditional Chinese snack dietary pattern, could improve clinical indicators of renal function [35].

The positive effects of MED on renal function are likely attributed to the combined effects of its various components. The MED components, such as vegetables, fruits, and nuts are rich in vitamins C and E, α-tocopherol, β-carotene, selenium, and polyphenols. These ingredients could effectively reduce the oxidative damage to lipids and proteins associated with hyperfiltration [36, 37]. Furthermore, a high MUFA/SFA ratio, coupled with omega-3 fatty acid content in fish could improve hyperlipidemia, endothelial function and creatinine-clearance rate and reduce blood pressure [38, 39]. Interestingly, our findings indicated that legumes are a pivotal component of MED reducing the risk of DKD. We hypothesize that dietary fiber derived from legumes could delay the increase in postprandial glycaemia and mitigate the risk factors associated with DKD, such as low-grade inflammation, hypertension, and hyperlipidemia [40, 41].

The strengths of this study included the long period of follow-up and the large sample size. To our knowledge, this is the first prospective cohort study that has examined the relationship between a MED and the incidence of microvascular complications among individuals with hyperglycemia. Furthermore, the analysis effectively addressed potential confounding effects by adjusting for several well-established circulating biomarkers. This is crucial as these biomarkers are positively correlated with an increased risk of DKD [42]. We also acknowledge several limitations. First, the identification of microvascular complications relied on hospital inpatient records and death registries. This may potentially result in an underestimation of cases, especially given the unavailability of complete primary care data at present. Second, among the T2DM patients, some individuals have information available on their diabetes duration, while others do not. It is challenging to adjust for diabetes duration in all T2DM patients. However, we conducted a sensitivity analysis by including the available diabetes duration data for those participants with this information. Our analysis revealed that the inclusion of the diabetes duration did not impact the main outcomes (Additional file 1: Table S10). Third, AMED may be associated with improvements in healthy lifestyle behaviours during follow-up, leading to improvements in HbA1c levels, blood pressure, lipid profile, and other relevant circulating biomarkers. However, data on these factors were only collected at recruitment in the UK Biobank [21, 43–45], making it challenging to assess the impact of their dynamic changes on the outcomes. Moreover, the impact of changes in the use of medications for diabetes, hyperlipemia, or hypertension on the main outcomes cannot be assessed due to the absence of data on this information during the 12.3-year follow-up period. Future studies with more detailed information during follow-up are required to verify our findings. Fourth, our study indicates a slightly lower incidence of diabetic retinopathy (11.3%) compared to nephropathy (13.3%) among hyperglycemic participants with T2DM. This is inconsistent with a previous population-based cohort study [46]. In our study, the primary outcomes predominantly derive from the inpatient registration system, rather than universal screening. Therefore, it is possible that fewer individuals are registered for retinopathy compared to nephropathy. Finally, we utilized dietary data from a self-administered 24-hour recall method. As multiple 24-hour recalls are necessary to obtain a 'true' representation of habitual diet, and considering that many participants in the UK Biobank completed only one or two recalls, it's possible that the calculated MED scores may not comprehensively reflect the usual dietary intake of the participants [47, 48]. However, as evidenced by other studies, individuals generally maintain a relatively stable dietary intake over time [49, 50] and thus it is less likely that their diet categorization will remarkably change.

## Conclusions

In this prospective cohort study, we have demonstrated that higher adherence to a MED is associated with a reduced risk of DKD among individuals with hyperglycemia. Our study emphasizes the necessity for continued research focusing on the benefits of the MED. Such efforts including the ongoing clinical trial will offer further insights into the role of MED in the clinical management of DKD.

### Supplementary Information

Additional file 1: Fig. S1. Dose-response relationship of AMED with the risks of microvascular complications among hyperglycemic participants without T2DM. The X-axis showed the numbers of AMED, and the y-axis showed the HRs of the composite microvascular complications (a), diabetic retinopathy (b), diabetic neuropathy (c), and diabetic kidney disease (d). Multivariable-adjusted models were adjusted for age (continuous, years) and gender (men or women), ethnicity (white or other), index of multiple deprivation (a measure of socioeconomic status), waist circumference (continuous, centimeters), alcohol consumption (categorized as never or special occasions only, one to three times a month, one to four times a week, daily or almost daily), physical activity (h/week), hypertension (yes or no), family history of diabetes (yes or no), family history of hypertension (yes or no), family history of heart disease (yes or no) and family history of stroke (yes or no), HbA1c (continuous, mmol/L), HDL-C (continuous, mmol/L), LDL-C (continuous, mmol/L), IGF (continuous, mmol/L), TG 30 (continuous, mmol/L), CHOL (continuous, mmol/L), use of antihypertensive medication and use of cholesterol lowering medication. Fig. S2. The association between adherence to individual components of AMED and the risks of microvascular complications among hyperglycemic participants without T2DM. One point was given for intakes above the median for fruit and vegetables, legumes and nuts, whole grains and fish. In addition, one point was given for intakes below the median of red and processed meat, for use of olive or rapeseed oil for cooking or as dressing and for moderate alcohol consumption with an average of 5–15 g of alcohol per day. Table S1. Components and scoring criteria of the Alternate Mediterranean Diet (AMED). Table S2. HRs (95% CIs) of microvascular complications according to the numbers of AMED among hyperglycemic participants without T2DM. Table S3. Stratified analyses of the associations of AMED with the risks of microvascular complications among total participants with hyperglycemia. Table S4. Stratified analyses of the associations of AMED with the risks of microvascular complications among hyperglycemic participants with T2DM. Table S5. Stratified analyses of the associations of AMED with the risks of microvascular complications among hyperglycemic participants without T2DM. Table S6. Sensitivity analyses of the associations between AMED scores and the risks of microvascular complications among total participants with hyperglycemia, after lagging the exposure for 2 or 4 years. Table S7. Sensitivity analyses of the associations between AMED scores and the risks of microvascular complications among hyperglycemic participants with T2DM, after lagging the exposure for 2 or 4 years. Table S8. Sensitivity analyses of the associations between AMED scores and the risks of microvascular complications among hyperglycemic participants without T2DM, after lagging the exposure for 2 or 4 years. Table S9. Sensitivity analyses of the associations between AMED scores and the risks of diabetic kidney disease (DKD) after including frequency of meat intake and creatinine as additional confounders in model 2 of DKD. Table S10. HRs (95% CIs) of microvascular complications according to the numbers of AMED among T2DM participants with available information on the diabetes duration.

## Data Availability

All data are available through request to the UK Biobank, and programs are available by contacting the corresponding author.

## Abbreviations

ACE: Angiotensin-converting enzyme
AMED: Alternate Mediterranean Diet
ARBs: Angiotensin-receptor blockers
CIs: Confidence intervals
CRT: Creatinine
DKD: Diabetic kidney disease
DN: Diabetic neuropathies
DR: Diabetic retinopathy
eGFR: Estimated glomerular filtration rate
GFR: Glomerular filtration rateGLP-1, glucagon-like peptide 1
HbA1c: Glycated hemoglobin
HDL-C: High-density lipoprotein cholesterol
HOMA-IR: Homeostatic Model Assessment for insulin resistance
HRs: Hazard ratios
IGF: Insulin-like growth factor 1
IMD: Index of Multiple Deprivation
LDL-C: Low-density lipoprotein cholesterol
MED: Mediterranean Diet
MUFA to SFA ratio: Monounsaturated-to-saturated fat ratio
MET: Metabolic equivalent
SGLT2: Sodium-glucose co-transporter-2
TC: Total cholesterol
TG: Triglycerides
T2DM: Type 2 diabetes mellitus
WC: Waist circumference
