# Supplementary material for: Adherence to a Mediterranean diet is associated with a lower risk of diabetic kidney disease among individuals with hyperglycemia: a prospective cohort study
Source: BMC Med. 2024 Jun 3;22:224. doi: 10.1186/s12916-024-03455-3 (PMC11149182; doi:10.1186/s12916-024-03455-3)
Supplement: Supplementary file 1 — Additional file 1: Fig. S1. Dose-response relationship of AMED with the risks of microvascular complications among hyperglycemic participants without T2DM. The X-axis showed the numbers of AMED, and the y-axis showed the HRs of the composite microvascular complications (a), diabetic retinopathy (b), diabetic neuropathy (c), and diabetic kidney disease (d). Multivariable-adjusted models were adjusted for age (continuous, years) and gender (men or women), ethnicity (white or other), index of multiple deprivation (a measure of socioeconomic status), waist circumference (continuous, centimeters), alcohol consumption (categorized as never or special occasions only, one to three times a month, one to four times a week, daily or almost daily), physical activity (h/week), hypertension (yes or no), family history of diabetes (yes or no), family history of hypertension (yes or no), family history of heart disease (yes or no) and family history of stroke (yes or no), HbA1c (continuous, mmol/L), HDL-C (continuous, mmol/L), LDL-C (continuous, mmol/L), IGF (continuous, mmol/L), TG 30 (continuous, mmol/L), CHOL (continuous, mmol/L), use of antihypertensive medication and use of cholesterol lowering medication. Fig. S2. The association between adherence to individual components of AMED and the risks of microvascular complications among hyperglycemic participants without T2DM. One point was given for intakes above the median for fruit and vegetables, legumes and nuts, whole grains and fish. In addition, one point was given for intakes below the median of red and processed meat, for use of olive or rapeseed oil for cooking or as dressing and for moderate alcohol consumption with an average of 5–15 g of alcohol per day. Table S1. Components and scoring criteria of the Alternate Mediterranean Diet (AMED). Table S2. HRs (95% CIs) of microvascular complications according to the numbers of AMED among hyperglycemic participants without T2DM. Table S3. Stratified analyses of the association [file 12916_2024_3455_MOESM1_ESM.zip › Additional file 1R5.docx]

Table S1. Components and scoring criteria of the Alternate Mediterranean Diet (AMED).

| **Component** | **Food** | **score of 1** | **Score of 0** |
| --- | --- | --- | --- |
| 1.Vegetables | Coleslaw, Side salad, Avocado, Beetroot, Broccoli, Butternut squash, Cabbage/kale,  Carrot, Cauliflower, Celery, Courgette, Cucumber, Garlic, Leek, Lettuce, Mushroom,  Onion, Parsnip, Sweet pepper, Spinach, Other vegetables | For each component, intakes above the median score 1 | For each component,  intakes below the  median score 0 |
| 2.Legumes | Baked bean, Pulses, Broad bean, Tofu |  |  |
| 3.Fruits | Fruit juice, Stewed fruit, Prune, Dried fruit, Mixed fruit, Apple, Banana, Berry,  Cherry, Grape, Mango, Melon, Orange, Satsuma, Other fruit |  |  |
| 4.Nuts | Salted peanuts, Unsalted peanuts, Salted nuts, Unsalted nuts, Seeds |  |  |
| 5.Whole grains | Muesli, Oat crunch, Sweetened cereal, Plain cereal, Bran cereal, Whole-wheat cereal,  Other cereal, Oatcakes, Wholemealpasta, Brown rice, Couscous, Cereal bar |  |  |
| 6.Fish | Tinned tuna, Oily fish, Breaded fish, Battered fish, White fish, Prawns, Lobster/crab,  Shellfish, Other fish |  |  |
| 7.MUFA:SFA  ratio | Monounsaturated fatty acids, Saturated fatty acids |  |  |
| 8.Red and  processed meat | Sausage, Beef, Pork, Lamb, Bacon, Ham, Liver | For each component, intakes below the median score 1 | For each component, intakes above the median score 0 |
| 9.Alcohol | Alcohol | 5 ≤ alcohol ≤ 15  g/d = 1 | alcohol <5 g/d or  > 15 g/d = 0 |
| Total score |  | 9 | 0 |

MUFA means monounsaturated fatty acids; SFA means saturated fatty acids.

| Table S2. HRs (95% CIs) of microvascular complications according to the numbers of AMED among hyperglycemic participants without T2DM. | | | | | | | | |
| --- | --- | --- | --- | --- | --- | --- | --- | --- |
|  | Numbers of AMED | | | | | | | |
|  | 0-1 | 2 | 3 | 4 | 5 | 6-9 | P _trend_ | HR _continuous_ |
| Number of patients | 2495 | 4425 | 5176 | 4878 | 4194 | 4304 |  |  |
| Composite microvascular complications | | | | | | |  |  |
| Cases/Person-years | 138/30687 | 273/54464 | 318/63726 | 273/60322 | 229/51868 | 222/53404 |  |  |
| Unadjusted | 1.00 | 1.12 (0.91, 1.38) | 1.10 (0.90, 1.34) | 0.97 (0.79, 1.19) | 0.93 (0.75, 1.14) | 0.88 (0.71, 1.08) | 0.005 | 0.96 (0.93, 0.99) |
| Model 1 | 1.00 | 1.12 (0.91, 1.37) | 1.11 (0.91, 1.35) | 0.98 (0.80, 1.20) | 0.94 (0.76, 1.16) | 0.89 (0.72, 1.11) | 0.012 | 0.97 (0.94, 0.99) |
| Model 2 | 1.00 | 1.15 (0.93, 1.41) | 1.18 (0.97, 1.45) | 1.09 (0.89, 1.34) | 1.05 (0.85, 1.30) | 1.04 (0.84, 1.29) | 0.479 | 0.99 (0.96, 1.02) |
| Diabetic retinopathy | | | | | | | | |
| Cases/Person-years | 18/31228 | 39/55455 | 35/65009 | 33/61398 | 31/52742 | 24/54303 |  |  |
| Unadjusted | 1.00 | 1.22 (0.70, 2.14) | 0.93 (0.53, 1.64) | 0.91 (0.51, 1.62) | 0.98 (0.55, 1.76) | 0.74 (0.40, 1.37) | 0.146 | 0.94 (0.86, 1.02) |
| Model 1 | 1.00 | 1.22 (0.70, 2.13) | 0.93 (0.52, 1.64) | 0.92 (0.52, 1.63) | 0.99 (0.56, 1.78) | 0.75 (0.41, 1.39) | 0.167 | 0.94 (0.86, 1.03) |
| Model 2 | 1.00 | 1.31 (0.75, 2.31) | 1.06 (0.60, 1.89) | 1.02 (0.57, 1.83) | 1.16 (0.64, 2.08) | 0.94 (0.51, 1.75) | 0.546 | 0.98 (0.90, 1.07) |
| Diabetic neuropathy | | | | | | | | |
| Cases/Person-years | 15/31225 | 36/55443 | 51/64937 | 51/61269 | 31/52699 | 41/54175 |  |  |
| Unadjusted | 1.00 | 1.36 (0.74, 2.48) | 1.62 (0.91, 2.89) | 1.70 (0.95, 3.02) | 1.18 (0.64, 2.19) | 1.53 (0.85, 2.76) | 0.483 | 1.03 (0.95, 1.11) |
| Model 1 | 1.00 | 1.35 (0.74, 2.46) | 1.66 (0.93, 2.94) | 1.75 (0.98, 3.10) | 1.22 (0.66, 2.26) | 1.61 (0.89, 2.91) | 0.333 | 1.04 (0.96, 1.12) |
| Model 2 | 1.00 | 1.38 (0.75, 2.52) | 1.77 (1.00, 3.16) | 1.93 (1.08, 3.44) | 1.38 (0.75, 2.57) | 1.90 (1.05, 3.45) | 0.080 | 1.07 (0.99, 1.16) |
| Diabetic kidney disease | | | | | | | | |
| Cases/Person-years | 110/30835 | 216/54692 | 249/64040 | 203/60650 | 176/52125 | 169/53677 |  |  |
| Unadjusted | 1.00 | 1.12 (0.89, 1.41) | 1.08 (0.86, 1.35) | 0.90 (0.71, 1.14) | 0.89 (0.70, 1.13) | 0.83 (0.65, 1.05) | 0.002 | 0.95 (0.92, 0.98) |
| Model 1 | 1.00 | 1.11 (0.88, 1.40) | 1.09 (0.87, 1.36) | 0.91 (0.72, 1.14) | 0.89 (0.70, 1.14) | 0.84 (0.66, 1.07) | 0.003 | 0.95 (0.92, 0.99) |
| Model 2 | 1.00 | 1.14 (0.91, 1.44) | 1.16 (0.92, 1.45) | 1.01 (0.80, 1.28) | 1.01 (0.79, 1.28) | 0.98 (0.77, 1.25) | 0.187 | 0.98 (0.95, 1.02) |

Model 1: age (continuous, years) and gender (men, women).

Model 2: Model 1+ ethnicity (white or other), index of multiple deprivation (a measure of socioeconomic status), waist circumference (continuous, in centimeters), alcohol consumption (categorized as never or special occasions only, one to three times a month, one to four times a week, daily or almost daily), physical activity (h/week), hypertension (yes or no), family history of diabetes (yes or no), family history of hypertension (yes or no), family history of heart disease(yes or no) and family history of stroke (yes or no), HbA1c (continuous, in mmol/L), HDL-C (continuous, in mmol/L), LDL-C (continuous, in mmol/L), IGF (continuous, in mmol/L), TG (continuous, in mmol/L), CHOL (continuous, in mmol/L), use of antihypertensive medication and use of cholesterol lowering medication. Composite microvascular complications refer to the development of any types of microvascular complications, including diabetic retinopathy, diabetic kidney disease, and diabetic neuropathy. We calculated person-years for these composite microvascular complications from the date of recruitment to the date of death, diagnosis of any microvascular complications, loss to follow-up, or the end of the follow-up period, whichever occurred first. The person-years for each specific outcome were computed individually without censoring other types of microvascular complications. CI, confidence interval.

Table S3. Stratified analyses of the associations of AMED with the risks of microvascular complications among total participants with hyperglycemia.

|  | Composite microvascular complications | Diabetic kidney disease | Diabetic retinopathy | Diabetic neuropathy |
| --- | --- | --- | --- | --- |
| Age, years | P-interaction = 0.01 | P-interaction = 0.001 | P-interaction = 0.364 | P-interaction = 0.023 |
| <55 | 0.91 (0.86, 0.97) | 0.84 (0.78, 0.92) | 0.96 (0.87, 1.05) | 0.96 (0.86, 1.07) |
| ≥55&<65 | 1.00 (0.97, 1.03) | 0.99 (0.96, 1.03) | 0.96 (0.92, 1.01) | 1.11 (1.04, 1.18) |
| ≥65 | 0.98 (0.94, 1.01) | 0.97 (0.93, 1.00) | 1.01 (0.95, 1.08) | 1.00 (0.91, 1.09) |
| Gender | P-interaction = 0.899 | P-interaction = 0.607 | P-interaction = 0.187 | P-interaction = 0.391 |
| Women | 0.98 (0.95, 1.02) | 0.96 (0.92, 1.00) | 1.02 (0.96, 1.08) | 1.08 (1.00, 1.17) |
| Men | 0.98 (0.95, 1.00) | 0.97 (0.94, 1.00) | 0.96 (0.91, 1.00) | 1.02 (0.97, 1.09) |
| IMD | P-interaction = 0.946 | P-interaction = 0.959 | P-interaction = 0.223 | P-interaction = 0.199 |
| <median (12.86) | 0.98 (0.95, 1.01) | 0.97 (0.93, 1.00) | 0.96 (0.90, 1.01) | 1.07 (1.00, 1.15) |
| ≥median (12.86) | 0.98 (0.95, 1.01) | 0.96 (0.93, 1.00) | 1.00 (0.95, 1.05) | 1.02 (0.96, 1.09) |
| WC, cm | P-interaction = 0.215 | P-interaction = 0.055 | P-interaction = 0.610 | P-interaction = 0.807 |
| Women<80, Men<90 | 0.96 (0.9, 1.02) | 0.90 (0.83, 0.98) | 0.99 (0.88, 1.10) | 1.06 (0.92, 1.23) |
| Women≥80, Men≥90 | 0.98 (0.96, 1.00) | 0.97 (0.95, 1.00) | 0.98 (0.94, 1.02) | 1.04 (0.99, 1.09) |
| Alcohol consumption | P-interaction = 0.432 | P-interaction = 0.507 | P-interaction = 0.258 | P-interaction = 0.675 |
| No | 0.96 (0.92, 1.00) | 0.97 (0.92, 1.02) | 0.94 (0.88, 1.01) | 1.02 (0.93, 1.12) |
| Yes | 0.99 (0.96, 1.01) | 0.96 (0.93, 0.99) | 0.99 (0.95, 1.04) | 1.05 (1.00, 1.11) |
| HbA1c, mmol/L | P-interaction = 0.593 | P-interaction = 0.419 | P-interaction = 0.889 | P-interaction = 0.988 |
| <48 | 0.99 (0.96, 1.01) | 0.97 (0.94, 1.00) | 0.99 (0.93, 1.05) | 1.04 (0.98, 1.11) |
| ≥48 | 0.97 (0.94, 1.01) | 0.95 (0.91, 1.00) | 0.98 (0.93, 1.03) | 1.05 (0.98, 1.13) |
| HDL-C, mmol/L | P-interaction = 0.737 | P-interaction = 0.734 | P-interaction = 0.955 | P-interaction = 0.836 |
| <1 | 0.97 (0.93, 1.01) | 0.96 (0.91, 1.01) | 0.97 (0.90, 1.06) | 1.05 (0.95, 1.16) |
| ≥1 | 0.98 (0.96, 1.00) | 0.97 (0.94, 1.00) | 0.98 (0.94, 1.02) | 1.04 (0.99, 1.10) |
| LDL-C, mmol/L | P-interaction = 0.061 | P-interaction = 0.345 | P-interaction = 0.566 | P-interaction = 0.075 |
| <3.4 | 0.99 (0.96, 1.01) | 0.97 (0.94, 1.00) | 0.98 (0.94, 1.02) | 1.07 (1.01, 1.13) |
| ≥3.4 | 0.96 (0.92, 0.99) | 0.95 (0.91, 1.00) | 0.98 (0.9, 1.07) | 0.98 (0.90, 1.07) |
| IGF, mmol/L | P-interaction = 0.735 | P-interaction = 0.731 | P-interaction = 0.409 | P-interaction = 0.791 |
| <median (20.95905) | 0.98 (0.95, 1.01) | 0.96 (0.93, 1.00) | 0.99 (0.94, 1.04) | 1.04 (0.97, 1.11) |
| ≥median (20.95905) | 0.98 (0.95, 1.01) | 0.97 (0.93, 1.00) | 0.96 (0.91, 1.02) | 1.05 (0.98, 1.13) |
| TG, mmol/L | P-interaction = 0.266 | P-interaction = 0.421 | P-interaction = 0.411 | P-interaction = 0.490 |
| <1.7 | 0.99 (0.96, 1.03) | 0.98 (0.94, 1.02) | 1.00 (0.95, 1.05) | 1.06 (0.98, 1.15) |
| ≥1.7 | 0.97 (0.94, 0.99) | 0.96 (0.93, 0.99) | 0.96 (0.92, 1.01) | 1.03 (0.98, 1.10) |
| TC, mmol/L | P-interaction = 0.118 | P-interaction = 0.486 | P-interaction = 0.449 | P-interaction = 0.308 |
| < 5.18 | 0.99 (0.96, 1.01) | 0.97 (0.94, 1.00) | 0.98 (0.94, 1.03) | 1.06 (1.00, 1.13) |
| ≥5.18 | 0.97 (0.94, 1.00) | 0.96 (0.92, 1.00) | 0.98 (0.91, 1.04) | 1.02 (0.95, 1.10) |
| Hypertension | P-interaction = 0.905 | P-interaction = 0.819 | P-interaction = 0.195 | P-interaction = 0.377 |
| No | 0.99 (0.93, 1.06) | 0.97 (0.88, 1.06) | 0.92 (0.82, 1.04) | 1.1 (0.95, 1.28) |
| Yes | 0.98 (0.96, 1.00) | 0.97 (0.94, 0.99) | 0.99 (0.95, 1.02) | 1.04 (0.99, 1.09) |
| Use of antihypertensive medication | P-interaction = 0.105 | P-interaction = 0.076 | P-interaction = 0.441 | P-interaction = 0.616 |
| No | 0.96 (0.93, 1.00) | 0.94 (0.90, 0.98) | 0.96 (0.91, 1.02) | 1.06 (0.99, 1.14) |
| Yes | 0.99 (0.96, 1.01) | 0.98 (0.95, 1.01) | 0.99 (0.94, 1.04) | 1.03 (0.97, 1.09) |
| Use of diabetes medication | P-interaction = 0.256 | P-interaction = 0.805 | P-interaction = 0.170 | P-interaction = 0.315 |
| No | 0.97 (0.95, 1.00) | 0.97 (0.94, 1.00) | 0.95 (0.89, 1.02) | 1.03 (0.96, 1.10) |
| Yes | 0.99 (0.96, 1.02) | 0.96 (0.92, 1.00) | 0.99 (0.95, 1.04) | 1.07 (1.00, 1.14) |
| Use of cholesterol lowering medication | P-interaction = 0.166 | P-interaction = 0.534 | P-interaction = 0.383 | P-interaction = 0.563 |
| No | 0.97 (0.94, 1.00) | 0.96 (0.92, 1.00) | 0.98 (0.91, 1.05) | 1.03 (0.96, 1.11) |
| Yes | 0.99 (0.96, 1.01) | 0.97 (0.94, 1.00) | 0.99 (0.94, 1.03 | 1.05 (0.99, 1.12) |
| Physical activity (MET), h/week | P-interaction = 0.494 | P-interaction = 0.655 | P-interaction = 0.754 | P-interaction = 0.070 |
| <median (26.125) | 0.97 (0.93, 1.00) | 0.96 (0.93, 0.99) | 0.97 (0.91, 1.02) | 1.01 (0.94, 1.08) |
| ≥median (26.125) | 0.97 (0.94, 1.01) | 0.95 (0.91, 0.99) | 0.98 (0.92, 1.04) | 1.08 (1.00, 1.17) |

Table S4. Stratified analyses of the associations of AMED with the risks of microvascular complications among hyperglycemic participants with T2DM.

|  | Composite microvascular complications | Diabetic kidney disease | Diabetic retinopathy | Diabetic neuropathy |
| --- | --- | --- | --- | --- |
| Age, years | P-interaction = 0.057 | P-interaction = 0.006 | P-interaction = 0.180 | P-interaction = 0.365 |
| <55 | 0.90 (0.83, 0.97) | 0.80 (0.70, 0.91) | 0.93 (0.84, 1.04) | 0.97 (0.84, 1.12) |
| ≥55&<65 | 1.00 (0.96, 1.04) | 1.00 (0.95, 1.05) | 0.98 (0.93, 1.04) | 1.07 (0.99, 1.16) |
| ≥65 | 0.97 (0.92, 1.01) | 0.94 (0.88, 0.99) | 1.03 (0.96, 1.11) | 1.01 (0.90, 1.14) |
| Gender | P-interaction = 0.333 | P-interaction = 0.990 | P-interaction = 0.022 | P-interaction = 0.040 |
| Women | 0.99 (0.95, 1.04) | 0.95 (0.89, 1.02) | 1.05 (0.99, 1.13) | 1.13 (1.02, 1.25) |
| Men | 0.96 (0.93, 1.00) | 0.95 (0.91, 0.99) | 0.95 (0.91, 1.00) | 0.99 (0.92, 1.06) |
| IMD | P-interaction = 0.808 | P-interaction = 0.900 | P-interaction = 0.934 | P-interaction = 0.419 |
| <median (12.86) | 0.98 (0.94, 1.01) | 0.95 (0.90, 1.00) | 0.99 (0.94, 1.05) | 1.05 (0.96, 1.15) |
| ≥median (12.86) | 0.97 (0.93, 1.01) | 0.95 (0.90, 1.00) | 0.99 (0.93, 1.05) | 1.02 (0.94, 1.11) |
| WC, cm | P-interaction = 0.632 | P-interaction = 0.657 | P-interaction = 0.786 | P-interaction = 0.814 |
| Women<80, Men<90 | 0.99 (0.89, 1.11) | 0.90 (0.75, 1.07) | 0.97 (0.84, 1.12) | 1.27 (0.96, 1.68) |
| Women≥80, Men≥90 | 0.97 (0.94, 1.00) | 0.95 (0.92, 0.99) | 0.99 (0.95, 1.03) | 1.02 (0.96, 1.09) |
| Alcohol consumption | P-interaction = 0.321 | P-interaction = 0.472 | P-interaction = 0.284 | P-interaction = 0.994 |
| No | 0.95 (0.90, 0.99) | 0.96 (0.90, 1.03) | 0.96 (0.89, 1.03) | 1.03 (0.93, 1.15) |
| Yes | 0.98 (0.95, 1.02) | 0.94 (0.90, 0.98) | 1.00 (0.96, 1.05) | 1.04 (0.97, 1.12) |
| HbA1c, mmol/mol | P-interaction = 0.831 | P-interaction = 0.695 | P-interaction = 0.377 | P-interaction = 0.399 |
| <48 | 0.97 (0.92, 1.01) | 0.94 (0.89, 1.00) | 1.01 (0.94, 1.08) | 0.98 (0.88, 1.09) |
| ≥48 | 0.98 (0.94, 1.01) | 0.96 (0.91, 1.00) | 0.98 (0.93, 1.03) | 1.06 (0.98, 1.14) |
| HDL-C, mmol/L | P-interaction = 0.965 | P-interaction = 0.940 | P-interaction = 0.750 | P-interaction = 0.930 |
| <1 | 0.97 (0.92, 1.02) | 0.95 (0.89, 1.02) | 0.97 (0.89, 1.06) | 1.02 (0.91, 1.14) |
| ≥1 | 0.97 (0.94, 1.01) | 0.95 (0.91, 0.99) | 0.99 (0.95, 1.04) | 1.04 (0.97, 1.11) |
| LDL-C, mmol/L | P-interaction = 0.111 | P-interaction = 0.749 | P-interaction = 0.837 | P-interaction = 0.060 |
| <3.4 | 0.98 (0.95, 1.01) | 0.95 (0.92, 0.99) | 0.99 (0.94, 1.03) | 1.06 (0.99, 1.13) |
| ≥3.4 | 0.91 (0.85, 0.98) | 0.92 (0.83, 1.03) | 0.99 (0.90, 1.10) | 0.90 (0.78, 1.05) |
| IGF, mmol/L | P-interaction = 0.783 | P-interaction = 0.595 | P-interaction = 0.833 | P-interaction = 0.820 |
| <median (20.95905) | 0.98 (0.94, 1.01) | 0.95 (0.90, 1.00) | 1.00 (0.94, 1.05) | 1.03 (0.94, 1.11) |
| ≥median (20.95905) | 0.97 (0.93, 1.01) | 0.95 (0.90, 0.99) | 0.99 (0.93, 1.04) | 1.04 (0.95, 1.13) |
| TG, mmol/L | P-interaction = 0.081 | P-interaction = 0.291 | P-interaction = 0.324 | P-interaction = 0.344 |
| <1.7 | 1.00 (0.96, 1.05) | 0.98 (0.92, 1.04) | 1.01 (0.95, 1.07) | 1.07 (0.96, 1.19) |
| ≥1.7 | 0.96 (0.92, 0.99) | 0.93 (0.89, 0.98) | 0.97 (0.92, 1.03) | 1.02 (0.95, 1.09) |
| TC, mmol/L | P-interaction = 0.242 | P-interaction = 0.722 | P-interaction = 0.773 | P-interaction = 0.388 |
| < 5.18 | 0.98 (0.95, 1.01) | 0.95 (0.91, 0.99) | 0.98 (0.94, 1.03) | 1.05 (0.97, 1.12) |
| ≥5.18 | 0.95 (0.89, 1.00) | 0.93 (0.86, 1.01) | 1.01 (0.93, 1.09) | 1.00 (0.89, 1.12) |
| Hypertension | P-interaction = 0.344 | P-interaction = 0.542 | P-interaction = 0.962 | P-interaction = 0.490 |
| No | 1.01 (0.91, 1.13) | 1.01 (0.85, 1.21) | 1.00 (0.86, 1.15) | 1.05 (0.85, 1.29) |
| Yes | 0.97 (0.94, 1.00) | 0.95 (0.91, 0.98) | 0.99 (0.95, 1.03) | 1.03 (0.97, 1.09) |
| Use of antihypertensive medication | P-interaction = 0.633 | P-interaction = 0.362 | P-interaction = 0.855 | P-interaction = 0.989 |
| No | 0.96 (0.92, 1.01) | 0.92 (0.85, 0.99) | 0.98 (0.92, 1.05) | 1.04 (0.94, 1.16) |
| Yes | 0.97 (0.94, 1.01) | 0.96 (0.92, 1.00) | 0.99 (0.94, 1.04) | 1.03 (0.96, 1.11) |
| Use of diabetes medication | P-interaction = 0.040 | P-interaction = 0.331 | P-interaction = 0.589 | P-interaction = 0.045 |
| No | 0.92 (0.87, 0.98) | 0.92 (0.86, 0.99) | 0.97 (0.88, 1.06) | 0.92 (0.80, 1.05) |
| Yes | 0.99 (0.96, 1.02) | 0.96 (0.92, 1.01) | 0.99 (0.95, 1.04) | 1.07 (1.00, 1.14) |
| Use of cholesterol lowering medication | P-interaction = 0.397 | P-interaction = 0.539 | P-interaction = 0.944 | P-interaction = 0.741 |
| No | 0.96 (0.90, 1.02) | 0.94 (0.87, 1.03) | 0.99 (0.90, 1.08) | 1.02 (0.90, 1.15) |
| Yes | 0.98 (0.95, 1.01) | 0.95 (0.92, 1.00) | 0.99 (0.95, 1.03) | 1.04 (0.97, 1.11) |
| Physical activity (MET), h/week | P-interaction = 0.822 | P-interaction = 0.766 | P-interaction = 0.576 | P-interaction = 0.051 |
| <median (26.125) | 0.96 (0.92, 1.00) | 0.96 (0.91, 1.02) | 0.98 (0.92, 1.04) | 0.98 (0.90, 1.07) |
| ≥median (26.125) | 0.97 (0.92, 1.01 | 0.92 (0.86, 0.98) | 0.98 (0.91, 1.04) | 1.07 (0.96, 1.18) |

Table S5. Stratified analyses of the associations of AMED with the risks of microvascular complications among hyperglycemic participants without T2DM.

|  | Composite microvascular complications | Diabetic kidney disease | Diabetic retinopathy | Diabetic neuropathy |
| --- | --- | --- | --- | --- |
| Age, years | P-interaction = 0.286 | P-interaction = 0.092 | P-interaction = 0.370 | P-interaction = 0.028 |
| <55 | 0.95 (0.88, 1.04) | 0.89 (0.80, 0.99) | 1.11 (0.92, 1.34) | 1.00 (0.84, 1.18) |
| ≥55&<65 | 1.01 (0.96, 1.05) | 0.99 (0.94, 1.04) | 0.92 (0.82, 1.04) | 1.17 (1.05, 1.31) |
| ≥65 | 0.99 (0.95, 1.04) | 1.00 (0.95, 1.05) | 0.99 (0.83, 1.17) | 0.97 (0.84, 1.12) |
| Gender | P-interaction = 0.237 | P-interaction = 0.351 | P-interaction = 0.449 | P-interaction = 0.304 |
| Women | 0.98 (0.93, 1.02) | 0.97 (0.92, 1.02) | 0.97 (0.85, 1.10) | 1.02 (0.90, 1.15) |
| Men | 1.01 (0.97, 1.05) | 0.99 (0.95, 1.04) | 1.01 (0.90, 1.14) | 1.10 (1.00, 1.21) |
| IMD | P-interaction = 0.762 | P-interaction = 0.882 | P-interaction = 0.730 | P-interaction = 0.575 |
| <median (12.86) | 1.00 (0.96, 1.04) | 0.99 (0.94, 1.04) | 0.97 (0.84, 1.11) | 1.08 (0.97, 1.21) |
| ≥median (12.86) | 0.99 (0.95, 1.03) | 0.98 (0.93, 1.02) | 0.98 (0.88, 1.10) | 1.06 (0.96, 1.18) |
| WC, cm | P-interaction = 0.056 | P-interaction = 0.030 | P-interaction = 0.706 | P-interaction = 0.417 |
| Women<80, Men<90 | 0.93 (0.86, 1.00) | 0.90 (0.82, 0.99) | 0.94 (0.76, 1.15) | 0.99 (0.83, 1.18) |
| Women≥80, Men≥90 | 1.00 (0.97, 1.04) | 0.99 (0.96, 1.03) | 0.94 (0.76, 1.15) | 1.09 (1.00, 1.18) |
| Alcohol consumption | P-interaction = 0.695 | P-interaction =0.964 | P-interaction = 0.629 | P-interaction = 0.367 |
| No | 0.98 (0.91, 1.04) | 0.97 (0.91, 1.04) | 0.92 (0.74, 1.14) | 1.02 (0.87, 1.20) |
| Yes | 1.00 (0.96, 1.03) | 0.98 (0.94, 1.02) | 0.99 (0.91, 1.09) | 1.08 (0.99, 1.18) |
| HbA1c, mmol/mol | P-interaction = 0.473 | P-interaction = 0.130 | P-interaction = 0.816 | P-interaction = 0.298 |
| <48 | 1.00 (0.97, 1.03) | 0.98 (0.95, 1.02) | 0.98 (0.88, 1.08) | 1.08 (1.00, 1.17) |
| ≥48 | 0.91 (0.77, 1.07) | 0.74 (0.51, 1.08) | 0.90 (0.74, 1.10) | 0.90 (0.51, 1.57) |
| HDL-C, mmol/L | P-interaction = 0.612 | P-interaction = 0.622 | P-interaction = 0.630 | P-interaction = 0.718 |
| <1 | 0.97 (0.90, 1.06) | 0.96 (0.88, 1.05) | 1.03 (0.80, 1.32) | 1.11 (0.90, 1.37) |
| ≥1 | 1.00 (0.97, 1.03) | 0.98 (0.95, 1.02) | 0.98 (0.89, 1.07) | 1.07 (0.98, 1.16) |
| LDL-C, mmol/L | P-interaction = 0.138 | P-interaction = 0.122 | P-interaction = 0.630 | P-interaction = 0.223 |
| <3.4 | 1.01 (0.97, 1.05) | 1.00 (0.96, 1.05) | 0.99 (0.89, 1.09) | 1.13 (1.01, 1.26) |
| ≥3.4 | 0.97 (0.93, 1.02) | 0.95 (0.90, 1.00) | 0.96 (0.81, 1.13) | 1.02 (0.92, 1.14) |
| IGF, mmol/L | P-interaction = 0.968 | P-interaction = 0.976 | P-interaction = 0.971 | P-interaction = 0.869 |
| <median (20.95905) | 0.99 (0.95, 1.03) | 0.98 (0.93, 1.03) | 0.98 (0.88, 1.09) | 1.07 (0.96, 1.19) |
| ≥median (20.95905) | 1.00 (0.95, 1.04) | 0.98 (0.94, 1.03) | 0.98 (0.85, 1.13) | 1.08 (0.97, 1.21) |
| TG, mmol/L | P-interaction = 0.985 | P-interaction = 0.892 | P-interaction = 0.834 | P-interaction = 0.911 |
| <1.7 | 0.99 (0.95, 1.04) | 0.98 (0.93, 1.04) | 0.99 (0.88, 1.12) | 1.06 (0.95, 1.20) |
| ≥1.7 | 0.99 (0.95, 1.03) | 0.98 (0.93, 1.02) | 0.93 (0.82, 1.06) | 1.08 (0.98, 1.20) |
| TC, mmol/L | P-interaction = 0.178 | P-interaction = 0.228 | P-interaction = 0.185 | P-interaction = 0.294 |
| < 5.18 | 1.01 (0.97, 1.06) | 1.00 (0.95, 1.06) | 1.02 (0.91, 1.15) | 1.13 (1.00, 1.28) |
| ≥5.18 | 0.98 (0.94, 1.02) | 0.96 (0.92, 1.01) | 0.93 (0.81, 1.06) | 1.04 (0.95, 1.15) |
| Hypertension | P-interaction = 0.568 | P-interaction =0.450 | P-interaction = 0.032 | P-interaction = 0.515 |
| No | 0.97 (0.89, 1.06) | 0.95 (0.85, 1.06) | 0.74 (0.58, 0.96) | 1.12 (0.90, 1.39) |
| Yes | 0.99 (0.96, 1.03) | 0.98 (0.95, 1.02) | 1.01 (0.93, 1.11) | 1.06 (0.98, 1.15) |
| Use of antihypertensive medication | P-interaction = 0.093 | P-interaction = 0.068 | P-interaction = 0.509 | P-interaction = 0.457 |
| No | 0.97 (0.93, 1.01) | 0.95 (0.90, 1.00) | 0.95 (0.85, 1.07) | 1.09 (0.99, 1.20) |
| Yes | 1.02 (0.97, 1.06) | 1.01 (0.96, 1.06) | 1.01 (0.88, 1.15) | 1.04 (0.92, 1.17) |
| Use of cholesterol lowering medication | P-interaction = 0.324 | P-interaction = 0.433 | P-interaction = 0.790 | P-interaction = 0.354 |
| No | 0.98 (0.94, 1.02) | 0.97 (0.93, 1.01) | 0.98 (0.87, 1.10) | 1.05 (0.96, 1.15) |
| Yes | 1.01 (0.96, 1.06) | 1.00 (0.95, 1.06) | 0.97 (0.86, 1.11) | 1.15 (0.99, 1.33) |
| Physical activity (MET), h/week | P-interaction = 0.438 | P-interaction = 0.497 | P-interaction = 0.655 | P-interaction = 0.994 |
| <median (26.125) | 0.98 (0.93, 1.02) | 0.96 (0.91, 1.02) | 0.95 (0.83, 1.07) | 1.08 (0.96, 1.2) |
| ≥median (26.125) | 0.99 (0.94, 1.04) | 0.97 (0.91, 1.02) | 0.98 (0.85, 1.14) | 1.11 (0.97, 1.27) |

Table S6. Sensitivity analyses of the associations between AMED scores and the risks of microvascular complications among total participants with hyperglycemia, after lagging the exposure for 2 or 4 years.

| Lagging the exposure (years) | Numbers of AMED | | | | | | | HR _continuous_ |
| --- | --- | --- | --- | --- | --- | --- | --- | --- |
|  | 0-1 | 2 | 3 | 4 | 5 | 6-9 | P _trend_ |  |
| Composite microvascular complications | | | | | | | | |
| 2 years | 1.00 | 0.98 (0.86, 1.12) | 0.98 (0.86, 1.11) | 0.97 (0.85, 1.10) | 0.92 (0.80, 1.05) | 0.88 (0.76, 1.01) | 0.029 | 0.98 (0.96, 1.00) |
| 4 years | 1.00 | 1.01 (0.88, 1.15) | 0.97 (0.85, 1.11) | 0.97 (0.85, 1.12) | 0.91 (0.79, 1.05) | 0.87 (0.75, 1.02) | 0.020 | 0.98 (0.96, 0.99) |
| Diabetic retinopathy | | | | | | | | |
| 2 years | 1.00 | 1.06 (0.84, 1.32) | 0.93 (0.74, 1.16) | 0.86 (0.68, 1.09) | 1.01 (0.80, 1.28) | 0.86 (0.67, 1.11) | 0.159 | 0.98 (0.94, 1.01) |
| 4 years | 1.00 | 1.07 (0.84, 1.36) | 0.93 (0.74, 1.18) | 0.89 (0.69, 1.14) | 1.03 (0.80, 1.32) | 0.87 (0.66, 1.14) | 0.234 | 0.98 (0.94, 1.02) |
| Diabetic neuropathy | | | | | | | | |
| 2 years | 1.00 | 1.10 (0.80, 1.51) | 1.16 (0.85, 1.58) | 1.29 (0.94, 1.77) | 1.07 (0.76, 1.50) | 1.38 (0.99, 1.93) | 0.090 | 1.05 (1.00, 1.10) |
| 4 years | 1.00 | 1.15 (0.82, 1.60) | 1.12 (0.81, 1.55) | 1.20 (0.86, 1.67) | 0.94 (0.66, 1.36) | 1.34 (0.95, 1.91) | 0.384 | 1.03 (0.97, 1.08) |
| Diabetic kidney disease | | | | | | | | |
| 2 years | 1.00 | 0.91 (0.78, 1.07) | 0.96 (0.82, 1.11) | 0.93 (0.79, 1.09) | 0.86 (0.73, 1.02) | 0.80 (0.67, 0.95) | 0.011 | 0.97 (0.94, 0.99) |
| 4 years | 1.00 | 0.94 (0.79, 1.10) | 0.94 (0.80, 1.11) | 0.95 (0.80, 1.12) | 0.88 (0.74, 1.05) | 0.80 (0.67, 0.96) | 0.017 | 0.97 (0.94, 1.00) |

Table S7. Sensitivity analyses of the associations between AMED scores and the risks of microvascular complications among hyperglycemic participants with T2DM, after lagging the exposure for 2 or 4 years.

| Lagging the exposure (years) | Numbers of AMED | | | | | | | HR _continuous_ |
| --- | --- | --- | --- | --- | --- | --- | --- | --- |
|  | 0-1 | 2 | 3 | 4 | 5 | 6-9 | P _trend_ |  |
| Composite microvascular complications | | | | | | | | |
| 2 years | 1.00 | 0.89 (0.75, 1.05) | 0.86 (0.73, 1.01) | 0.92 (0.78, 1.09) | 0.87 (0.73, 1.03) | 0.78 (0.65, 0.94) | 0.041 | 0.97 (0.95, 1.00) |
| 4 years | 1.00 | 0.91 (0.76, 1.08) | 0.87 (0.73, 1.04) | 0.92 (0.77, 1.10) | 0.86 (0.71, 1.04) | 0.80 (0.65, 0.97) | 0.049 | 0.97 (0.94, 1.00) |
| Diabetic retinopathy | | | | | | | | |
| 2 years | 1.00 | 1.00 (0.78, 1.27) | 0.91 (0.71, 1.16) | 0.86 (0.67, 1.11) | 1.03 (0.80, 1.34) | 0.88 (0.66, 1.16) | 0.460 | 0.99 (0.95, 1.03) |
| 4 years | 1.00 | 1.00 (0.77, 1.30) | 0.92 (0.71, 1.19) | 0.88 (0.67, 1.16) | 1.07 (0.81, 1.41) | 0.89 (0.66, 1.20) | 0.657 | 0.99 (0.95, 1.04) |
| Diabetic neuropathy | | | | | | | | |
| 2 years | 1.00 | 0.97 (0.67, 1.41) | 0.91 (0.63, 1.32) | 1.07 (0.73, 1.55) | 0.94 (0.63, 1.41) | 1.19 (0.80, 1.79) | 0.397 | 1.03 (0.97, 1.10) |
| 4 years | 1.00 | 1.00 (0.68, 1.48) | 0.89 (0.61, 1.32) | 0.98 (0.66, 1.46) | 0.82 (0.53, 1.27) | 1.18 (0.78, 1.80) | 0.801 | 1.01 (0.95, 1.08) |
| Diabetic kidney disease | | | | | | | | |
| 2 years | 1.00 | 0.76 (0.61, 0.95) | 0.81 (0.66, 0.99) | 0.88 (0.71, 1.09) | 0.76 (0.60, 0.96) | 0.65 (0.51, 0.84) | 0.017 | 0.95 (0.92, 0.99) |
| 4 years | 1.00 | 0.78 (0.62, 0.98) | 0.81 (0.65, 1.01) | 0.89 (0.71, 1.11) | 0.78 (0.61, 0.99) | 0.67 (0.51, 0.87) | 0.032 | 0.95 (0.92, 0.99) |

Table S8. Sensitivity analyses of the associations between AMED scores and the risks of microvascular complications among hyperglycemic participants without T2DM, after lagging the exposure for 2 or 4 years.

| Lagging the exposure (years) | Numbers of AMED | | | | | | | HR _continuous_ |
| --- | --- | --- | --- | --- | --- | --- | --- | --- |
|  | 0-1 | 2 | 3 | 4 | 5 | 6-9 | P _trend_ |  |
| Composite microvascular complications | | | | | | | | |
| 2 years | 1.00 | 1.13 (0.92, 1.39) | 1.18 (0.96, 1.44) | 1.08 (0.88, 1.33) | 1.05 (0.84, 1.30) | 1.05 (0.84, 1.30) | 0.570 | 1.00 (0.97, 1.03) |
| 4 years | 1.00 | 1.16 (0.94, 1.44) | 1.12 (0.91, 1.39) | 1.10 (0.89, 1.37) | 1.03 (0.82, 1.29) | 1.01 (0.80, 1.26) | 0.348 | 0.99 (0.96, 1.02) |
| Diabetic retinopathy | | | | | | | | |
| 2 years | 1.00 | 1.33 (0.76, 2.33) | 1.01 (0.57, 1.80) | 0.99 (0.55, 1.77) | 1.11 (0.61, 2.02) | 0.95 (0.51, 1.76) | 0.502 | 0.97 (0.89, 1.06) |
| 4 years | 1.00 | 1.37 (0.77, 2.44) | 0.94 (0.51, 1.71) | 1.05 (0.58, 1.89) | 1.05 (0.57, 1.95) | 0.92 (0.48, 1.74) | 0.398 | 0.97 (0.88, 1.06) |
| Diabetic neuropathy | | | | | | | | |
| 2 years | 1.00 | 1.46 (0.77, 2.77) | 1.92 (1.04, 3.54) | 2.09 (1.13, 3.87) | 1.54 (0.80, 2.96) | 2.07 (1.10, 3.90) | 0.052 | 1.08 (1.00, 1.17) |
| 4 years | 1.00 | 1.57 (0.81, 3.05) | 1.82 (0.96, 3.45) | 1.97 (1.04, 3.76) | 1.39 (0.70, 2.76) | 1.95 (1.01, 3.79) | 0.180 | 1.06 (0.97, 1.15) |
| Diabetic kidney disease | | | | | | | | |
| 2 years | 1.00 | 1.11 (0.88, 1.40) | 1.15 (0.92, 1.44) | 1.01 (0.80, 1.28) | 0.99 (0.78, 1.27) | 0.98 (0.77, 1.24) | 0.233 | 0.98 (0.95, 1.02) |
| 4 years | 1.00 | 1.13 (0.89, 1.45) | 1.11 (0.87, 1.41) | 1.05 (0.82, 1.33) | 1.01 (0.78, 1.29) | 0.96 (0.74, 1.24) | 0.226 | 0.98 (0.95, 1.02) |

Table S9. Sensitivity analyses of the associations between AMED scores and the risks of diabetic kidney disease (DKD) after including frequency of meat intake and creatinine as additional confounders in model 2 of DKD.

|  | Numbers of AMED | | | | | | | HR _continuous_ |
| --- | --- | --- | --- | --- | --- | --- | --- | --- |
|  | 0-1 | 2 | 3 | 4 | 5 | 6-9 | P _trend_ |  |
| Participants with hyperglycemia | | | | | | | | |
| Model 2 + frequency of meat intake | 1.00 | 0.92 (0.79, 1.08) | 0.96 (0.82, 1.11) | 0.92 (0.79, 1.08) | 0.86 (0.73, 1.01) | 0.79 (0.67, 0.94) | 0.005 | 0.97 (0.94, 0.99) |
| Model 2 + creatinine | 1.00 | 0.90 (0.77, 1.05) | 0.89 (0.76, 1.04) | 0.92 (0.79, 1.08) | 0.87 (0.74, 1.02) | 0.80 (0.68, 0.95) | 0.033 | 0.97 (0.95, 0.99) |
| Hyperglycemic participants with T2DM | | | | | | | | |
| Model 2 + frequency of meat intake | 1.00 | 0.76 (0.62, 0.94) | 0.81 (0.66, 0.99) | 0.86 (0.70, 1.06) | 0.76 (0.60, 0.95) | 0.64 (0.50, 0.82) | 0.009 | 0.95 (0.91, 0.99) |
| Model 2 + creatinine | 1.00 | 0.78 (0.63, 0.96) | 0.78 (0.63, 0.96) | 0.88 (0.71, 1.08) | 0.78 (0.62, 0.98) | 0.69 (0.54, 0.89) | 0.049 | 0.96 (0.92, 0.99) |
| Hyperglycemic participants without T2DM | | | | | | | | |
| Model 2 + frequency of meat intake | 1.00 | 1.14 (0.90, 1.43) | 1.15 (0.92, 1.44) | 1.02 (0.80, 1.28) | 1.00 (0.78, 1.26) | 0.97 (0.76, 1.24) | 0.169 | 0.98 (0.95, 1.01) |
| Model 2 + creatinine | 1.00 | 1.10 (0.88, 1.39) | 1.07 (0.85, 1.34) | 1.02 (0.80, 1.28) | 1.01 (0.79, 1.28) | 0.98 (0.77, 1.25) | 0.402 | 0.99 (0.96, 1.02) |

* Based on the confounders in model 2 as shown in table 2, the creatinine levels (continuous, µmol/L) and frequency of meat intake (times/week) were additionally adjusted for participants diagnosed with DKD.

| Table S10. HRs (95% CIs) of microvascular complications according to the numbers of AMED among T2DM participants with available information on the diabetes duration. | | | | | | | | |
| --- | --- | --- | --- | --- | --- | --- | --- | --- |
|  | Numbers of AMED | | | | | | | |
|  | 0-1 | 2 | 3 | 4 | 5 | 6-9 | P _trend_ | HR _continuous_ |
| Number of patients | 434 | 843 | 917 | 765 | 592 | 511 |  |  |
| Composite microvascular complications | | | | | | |  |  |
| Cases/Person-years | 121/4718 | 219/9406 | 222/10344 | 191/8706 | 135/6714 | 109/5905 |  |  |
| Unadjusted | 1.00 | 0.89 (0.72, 1.12) | 0.81 (0.65, 1.01) | 0.83 (0.66, 1.04) | 0.75 (0.59, 0.96) | 0.67 (0.52, 0.87) | 0.001 | 0.94 (0.90, 0.98) |
| Model 1 | 1.00 | 0.89 (0.72, 1.12) | 0.81 (0.65, 1.01) | 0.83 (0.66, 1.05) | 0.75 (0.59, 0.96) | 0.68 (0.52, 0.88) | 0.001 | 0.94 (0.90, 0.98) |
| Model 2 | 1.00 | 0.90 (0.72, 1.12) | 0.81 (0.65, 1.01) | 0.88 (0.70, 1.11) | 0.76 (0.60, 0.98) | 0.75 (0.58, 0.98) | 0.021 | 0.96 (0.92, 0.99) |
| Model 3 | 1.00 | 0.90 (0.72, 1.12) | 0.81 (0.65, 1.02) | 0.89 (0.71, 1.12) | 0.76 (0.60, 0.98) | 0.75 (0.58, 0.97) | 0.020 | 0.96 (0.92, 0.99) |
| Diabetic retinopathy | | | | | | | | |
| Cases/Person-years | 49/5164 | 98/10042 | 99/11043 | 73/9357 | 62/7118 | 50/6202 |  |  |
| Unadjusted | 1.00 | 1.02 (0.72, 1.43) | 0.92 (0.66, 1.30) | 0.80 (0.56, 1.15) | 0.90 (0.62, 1.30) | 0.81 (0.55, 1.20) | 0.124 | 0.96 (0.91, 1.02) |
| Model 1 | 1.00 | 1.02 (0.72, 1.44) | 0.93 (0.66, 1.31) | 0.81 (0.56, 1.16) | 0.89 (0.61, 1.30) | 0.82 (0.55, 1.22) | 0.136 | 0.96 (0.91, 1.02) |
| Model 2 | 1.00 | 0.98 (0.69, 1.38) | 0.91 (0.64, 1.28) | 0.86 (0.60, 1.23) | 0.93 (0.63, 1.35) | 0.91 (0.61, 1.35) | 0.503 | 0.98 (0.93, 1.04) |
| Model 3 | 1.00 | 0.97 (0.69, 1.38) | 0.91 (0.64, 1.28) | 0.86 (0.60, 1.24) | 0.92 (0.63, 1.34) | 0.90 (0.60, 1.34) | 0.503 | 0.98 (0.93, 1.04) |
| Diabetic neuropathy | | | | | | | | |
| Cases/Person-years | 28/5259 | 49/10325 | 51/11254 | 43/9439 | 33/7259 | 23/6311 |  |  |
| Unadjusted | 1.00 | 0.89 (0.56, 1.41) | 0.85 (0.53, 1.34) | 0.85 (0.53, 1.36) | 0.84 (0.51, 1.39) | 0.67 (0.38, 1.16) | 0.198 | 0.94 (0.87, 1.02) |
| Model 1 | 1.00 | 0.89 (0.56, 1.41) | 0.85 (0.54, 1.35) | 0.85 (0.53, 1.37) | 0.84 (0.51, 1.39) | 0.68 (0.39, 1.18) | 0.212 | 0.95 (0.87, 1.03) |
| Model 2 | 1.00 | 0.85 (0.53, 1.35) | 0.83 (0.52, 1.33) | 0.90 (0.56, 1.45) | 0.85 (0.51, 1.40) | 0.78 (0.45, 1.36) | 0.552 | 0.97 (0.90, 1.05) |
| Model 3 | 1.00 | 0.85 (0.53, 1.35) | 0.85 (0.53, 1.35) | 0.92 (0.57, 1.48) | 0.86 (0.52, 1.42) | 0.78 (0.45, 1.36) | 0.581 | 0.97 (0.90, 1.06) |
| Diabetic kidney disease | | | | | | | | |
| Cases/Person-years | 79/5013 | 117/9992 | 134/10852 | 112/9086 | 74/7124 | 63/6173 |  |  |
| Unadjusted | 1.00 | 0.73 (0.55, 0.98) | 0.75 (0.57, 0.99) | 0.75 (0.56, 1.00) | 0.62 (0.45, 0.86) | 0.59 (0.42, 0.82) | 0.003 | 0.92 (0.88, 0.97) |
| Model 1 | 1.00 | 0.73 (0.55, 0.98) | 0.76 (0.57, 1.00) | 0.76 (0.57, 1.01) | 0.62 (0.45, 0.85) | 0.60 (0.43, 0.83) | 0.003 | 0.93 (0.88, 0.97) |
| Model 2 | 1.00 | 0.74 (0.55, 0.98) | 0.75 (0.57, 0.99) | 0.79 (0.59, 1.05) | 0.64 (0.46, 0.88) | 0.67 (0.48, 0.94) | 0.027 | 0.94 (0.90, 0.99) |
| Model 3 | 1.00 | 0.74 (0.55, 0.98) | 0.75 (0.57, 0.99) | 0.79 (0.59, 1.06) | 0.64 (0.46, 0.87) | 0.67 (0.48, 0.94) | 0.027 | 0.94 (0.90, 0.99) |

Model 1: age (continuous, years) and sex (men, women).

Model 2: Model 1+ ethnicity (white or other), index of multiple deprivation (a measure of socioeconomic status), waist circumference (continuous, in centimeters), alcohol consumption (categorized as never or special occasions only, one to three times a month, one to four times a week, daily or almost daily), physical activity (h/week), hypertension (yes or no), family history of diabetes (yes or no), family history of hypertension (yes or no), family history of heart disease(yes or no) and family history of stroke (yes or no), HbA1c (continuous, in mmol/L), HDL-C (continuous, in mmol/L), LDL-C (continuous, in mmol/L), IGF (continuous, in mmol/L), TG (continuous, in mmol/L), CHOL (continuous, in mmol/L), use of antihypertensive medication, use of cholesterol lowering medication and use of diabetes medication. Composite microvascular complications refer to the development of any types of microvascular complications, including diabetic retinopathy, diabetic kidney disease, and diabetic neuropathy. We calculated person-years for these composite microvascular complications from the date of recruitment to the date of death, diagnosis of any microvascular complications, loss to follow-up, or the end of the follow-up period, whichever occurred first. The person-years for each specific outcome were computed individually without censoring other types of microvascular complications. CI, confidence interval.

Model 3: Model 1 + Model2 + diabetes duration.
