# Supplementary figures and images for: Adherence to a Mediterranean diet is associated with a lower risk of diabetic kidney disease among individuals with hyperglycemia: a prospective cohort study
Source: BMC Med. 2024 Jun 3;22:224. doi: 10.1186/s12916-024-03455-3 (PMC11149182; doi:10.1186/s12916-024-03455-3)

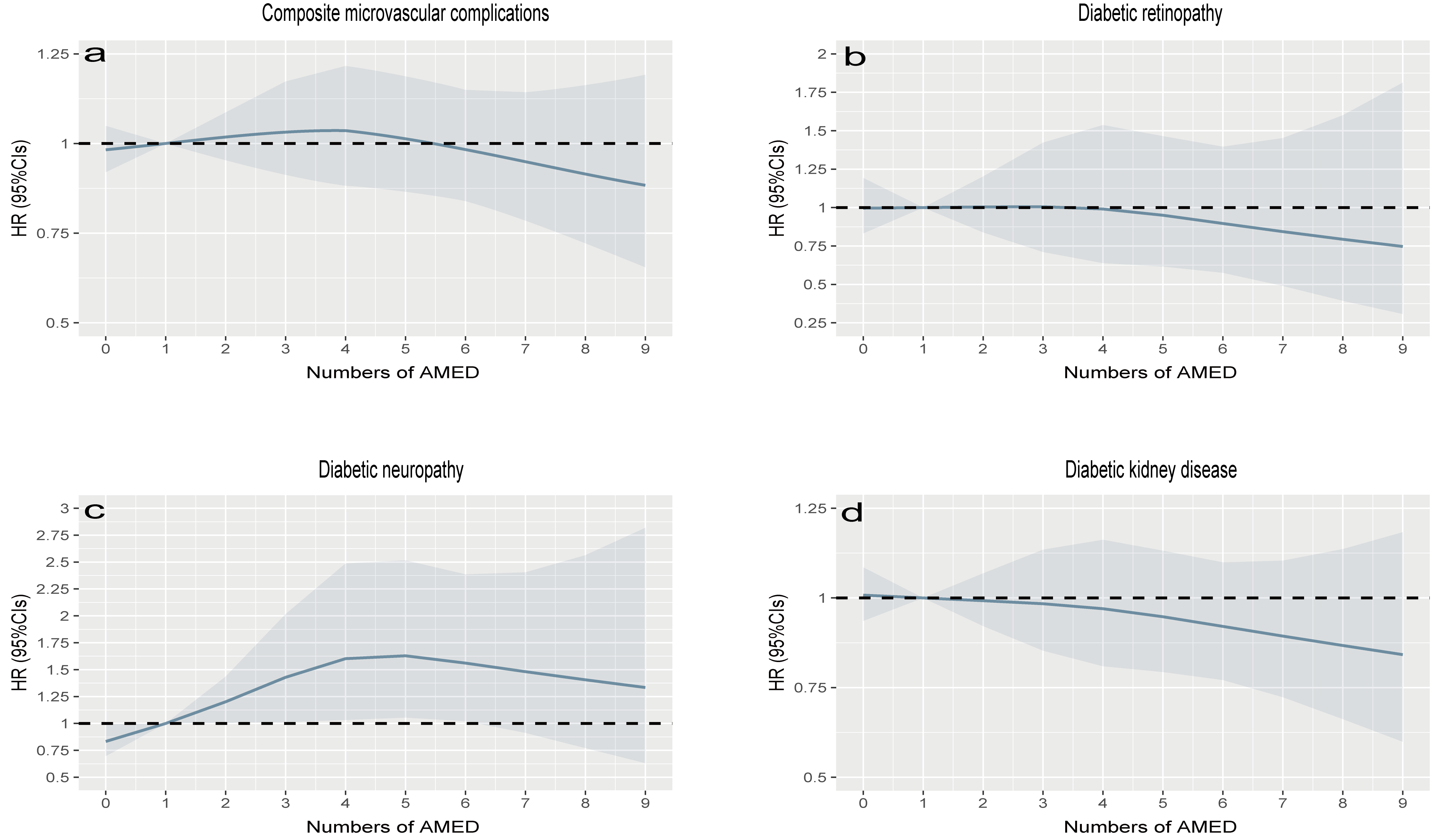

Supplement: Supplementary file 1 — Additional file 1: Fig. S1. Dose-response relationship of AMED with the risks of microvascular complications among hyperglycemic participants without T2DM. The X-axis showed the numbers of AMED, and the y-axis showed the HRs of the composite microvascular complications (a), diabetic retinopathy (b), diabetic neuropathy (c), and diabetic kidney disease (d). Multivariable-adjusted models were adjusted for age (continuous, years) and gender (men or women), ethnicity (white or other), index of multiple deprivation (a measure of socioeconomic status), waist circumference (continuous, centimeters), alcohol consumption (categorized as never or special occasions only, one to three times a month, one to four times a week, daily or almost daily), physical activity (h/week), hypertension (yes or no), family history of diabetes (yes or no), family history of hypertension (yes or no), family history of heart disease (yes or no) and family history of stroke (yes or no), HbA1c (continuous, mmol/L), HDL-C (continuous, mmol/L), LDL-C (continuous, mmol/L), IGF (continuous, mmol/L), TG 30 (continuous, mmol/L), CHOL (continuous, mmol/L), use of antihypertensive medication and use of cholesterol lowering medication. Fig. S2. The association between adherence to individual components of AMED and the risks of microvascular complications among hyperglycemic participants without T2DM. One point was given for intakes above the median for fruit and vegetables, legumes and nuts, whole grains and fish. In addition, one point was given for intakes below the median of red and processed meat, for use of olive or rapeseed oil for cooking or as dressing and for moderate alcohol consumption with an average of 5–15 g of alcohol per day. Table S1. Components and scoring criteria of the Alternate Mediterranean Diet (AMED). Table S2. HRs (95% CIs) of microvascular complications according to the numbers of AMED among hyperglycemic participants without T2DM. Table S3. Stratified analyses of the association [file 12916_2024_3455_MOESM1_ESM.zip › Fig.S1R5.tif]

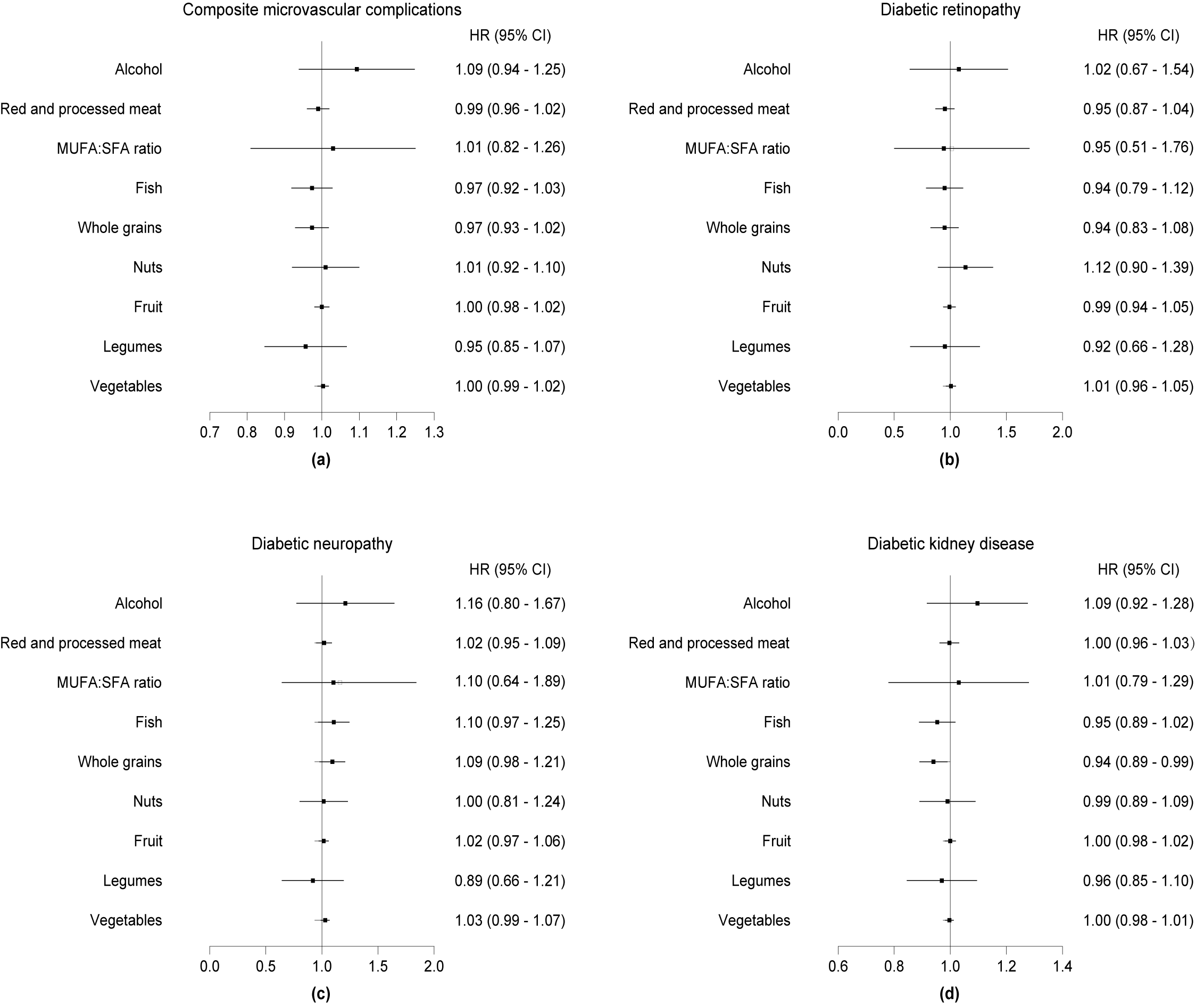

Supplement: Supplementary file 1 — Additional file 1: Fig. S1. Dose-response relationship of AMED with the risks of microvascular complications among hyperglycemic participants without T2DM. The X-axis showed the numbers of AMED, and the y-axis showed the HRs of the composite microvascular complications (a), diabetic retinopathy (b), diabetic neuropathy (c), and diabetic kidney disease (d). Multivariable-adjusted models were adjusted for age (continuous, years) and gender (men or women), ethnicity (white or other), index of multiple deprivation (a measure of socioeconomic status), waist circumference (continuous, centimeters), alcohol consumption (categorized as never or special occasions only, one to three times a month, one to four times a week, daily or almost daily), physical activity (h/week), hypertension (yes or no), family history of diabetes (yes or no), family history of hypertension (yes or no), family history of heart disease (yes or no) and family history of stroke (yes or no), HbA1c (continuous, mmol/L), HDL-C (continuous, mmol/L), LDL-C (continuous, mmol/L), IGF (continuous, mmol/L), TG 30 (continuous, mmol/L), CHOL (continuous, mmol/L), use of antihypertensive medication and use of cholesterol lowering medication. Fig. S2. The association between adherence to individual components of AMED and the risks of microvascular complications among hyperglycemic participants without T2DM. One point was given for intakes above the median for fruit and vegetables, legumes and nuts, whole grains and fish. In addition, one point was given for intakes below the median of red and processed meat, for use of olive or rapeseed oil for cooking or as dressing and for moderate alcohol consumption with an average of 5–15 g of alcohol per day. Table S1. Components and scoring criteria of the Alternate Mediterranean Diet (AMED). Table S2. HRs (95% CIs) of microvascular complications according to the numbers of AMED among hyperglycemic participants without T2DM. Table S3. Stratified analyses of the association [file 12916_2024_3455_MOESM1_ESM.zip › Fig.S2R5.tif]
